# Response of Midgut Trypsin- and Chymotrypsin-Like Proteases of *Helicoverpa armigera* Larvae Upon Feeding With Peanut BBI: Biochemical and Biophysical Characterization of PnBBI

**DOI:** 10.3389/fpls.2020.00266

**Published:** 2020-03-24

**Authors:** Vadthya Lokya, Marri Swathi, Nalini Mallikarjuna, Kollipara Padmasree

**Affiliations:** ^1^Department of Biotechnology and Bioinformatics, School of Life Sciences, University of Hyderabad, Hyderabad, India; ^2^Department of Plant Sciences, School of Life Sciences, University of Hyderabad, Hyderabad, India; ^3^Legumes Cell Biology, Grain Legumes Program, ICRISAT, Hyderabad, India

**Keywords:** *Arachis hypogaea* (Fabaceae), PnBBI, two-dimensional zymography, circular dichroism, surface plasmon resonance, *Helicoverpa armigera* (Noctuidae), trypsin-like midgut proteases

## Abstract

Proteinase/Protease inhibitors (PIs) from higher plants play an important role in defense and confer resistance against various insect pests and pathogens. In the present study, Bowman-Birk Inhibitor (BBI) was purified from mature seeds of an interspecific advanced hybrid peanut variety (4368-1) using chromatographic techniques. The biochemical and biophysical characteristics such as low molecular mass, presence of several isoinhibitors and higher-ordered dimer/tetramer, predominance of antiparallel β-sheets and random coils in secondary structure, reactive sites against trypsin and chymotrypsin, broad spectrum of stability toward extreme pH and temperature along with MALDI TOF-TOF analysis (ProteomeXchange identifier PXD016933) ascertained the purified biomolecule from peanut as BBI (PnBBI). Surface plasmon resonance competitive binding analysis revealed the bifunctional PnBBI is a trypsin specific inhibitor with 1:2 stoichiometry as compared to chymotrypsin. A concentration-dependent self-association tendency of PnBBI was further confirmed by ‘red shift’ in the far-UV CD spectra. Furthermore, the insecticidal potential of PnBBI against *Helicoverpa armigera* was assessed by *in vitro* assays and *in vivo* feeding experiments. A significant reduction in larval body weight was observed with concomitant attenuation in the activity of midgut trypsin-like proteases of *H. armigera* (HaTPs) fed on PnBBI supplemented diet. The one and two-dimensional zymography studies revealed the disappearance of several isoforms of HaTP upon feeding with PnBBI. qRT-PCR analysis further suggests the role of PnBBI in not only inhibiting the activity of midgut trypsin and chymotrypsin-like proteases but also in modulating their expression. Taken together, the results provide a biochemical and molecular basis for introgressed resistance in peanut interspecific advanced hybrid variety against *H. armigera*.

## Introduction

Proteolysis is a key physiological process catalyzed by specific proteases in plants, animals and microorganisms (Lopez-Otin and Bond, 2009). The exogenous and endogenous proteolytic activity of four mechanistic classes (serine, cysteine, aspartate, and metallo-) of proteases are highly regulated by corresponding proteinase/protease inhibitors (PIs). In higher plants, PIs are constitutively expressed in storage organs and induced in vegetative organs in response to wounding/herbivory during the invasion of microbial pathogens or infestation by insect pests (Ryan, 1990; Shewry, 2003; Rehman et al., 2017). On the other hand, PIs are known to play a role in the regulation of programed cell death and tolerance against abiotic stress (Drame et al., 2013; Boex-fontvieille et al., 2015). Several studies demonstrated the use of PIs in the management of agriculturally important insect pests (Shamsi et al., 2016; War et al., 2018; Clemente et al., 2019). In this regard, serine PIs such as Kunitz and Bowman-Birk inhibitors (BBIs) are receiving considerable attention as a part of Integrated Pest Management (IPM) since lepidopteran insect pests depend predominantly on trypsin- and chymotrypsin-like digestive proteases for attaining energy (Sharma et al., 2000; Srinivasan et al., 2006; Swathi et al., 2016). The Kunitz PIs possess typical characteristics such as molecular mass of 14-24 kDa, single polypeptide chain containing one reactive site with β-trefoil structure stabilized by two disulfide bonds and inhibit serine, cysteine and aspartic proteases (Bendre et al., 2018). BBIs are ∼8 kDa proteins with two reactive sites specific for trypsin and chymotrypsin which are stabilized by seven intramolecular disulfide bonds (Macedo et al., 2015). These canonical serine PIs follows the standard mechanism of inhibition by interacting with the active site of proteases by tight binding reaction (Laskowski and Qasim, 2000; Bateman and James, 2011).

*Helicoverpa armigera* (Lepidoptera: Noctuidae) is one among the devastating insect pests of agriculture, globally accounting to an estimated loss of US $ 2 billion annually (Tay et al., 2013; EPPO, 2019). It infests more than 200 plant species including economically important agricultural crops such as cotton, tomato, pigeon pea, chickpea, sorghum, cowpea, maize, tobacco, and peanut (Pratissoli et al., 2015). The direct feeding habit of *H. armigera* larvae on flowering and fruiting bodies of the plant is cited as an important rationale for a significant loss in crop yield (McGahan et al., 1991). Though various pesticides from both synthetic and biochemical origin are employed, control of *H. armigera* invasion remained challenging due to its survival and adaptability to unstable habitats and seasonable changes (Fitt, 1989; Tabashnik and Carrière, 2017). The enormous flexibility in the expression of gut proteases of *H. armigera* upon feeding on different protein-rich parts of the plant allowed it to adapt and augment polyphagous nature (Patankar et al., 2001; Chikate et al., 2013; Macedo et al., 2015). The expression of larval midgut proteases switches among several gene copies of serine, cysteine, aminopeptidases depending on their developmental stage and diet composition (Sarate et al., 2012; Kipgen and Aggarwal, 2014). In addition, insects use different defensive strategies to minimize the anti-nutritional effect of ingested plant PIs such as (i) compensatory overproduction of existing digestive proteases, (ii) expression of inhibitor insensitive digestive proteases and (iii) production of PI hydrolyzing proteases (Bown et al., 1997; Jongsma and Bolter, 1997; Girard et al., 1998; Zhu-Salzman et al., 2003; Macedo et al., 2015). Further, the extensive usage of pesticides causes deleterious effects on natural ecosystem such as loss of biodiversity, human health hazards, pest resistance and a surge of secondary pests, ultimately leading to outbursts of pests (Aktar et al., 2009; Nicolopoulou-Stamati et al., 2016). Therefore, it is necessary to develop novel eco-friendly strategies such as application of PIs to manage the damage caused by *H. armigera* and reduce the hazardous pesticide use including organophosphates, synthetic pyrethroids, carbamates and endosulfan (Sharma et al., 2000; Downes et al., 2017). In general, the PIs bind firmly and irreversibly to the active site of digestive enzymes and attenuate their activity, which in turn impair the protein turnover in various metabolic processes, eventually limits their growth and development (Zhu-Salzman and Zeng, 2015). Also, the resistance mediated by PIs from wild-relatives or non-host or hybrid plants is more advantageous than host-plant resistance, due to inexposure of pest midgut proteases to PIs from such plants (Parde et al., 2010; Pandey et al., 2014; Swathi et al., 2016; de Paula et al., 2017).

Peanut (*Arachis hypogaea* L.) is one of the important oilseed legume crops of tropical and semi-arid tropical countries of Asia, Africa, and America (Arya et al., 2016). The cultivated varieties of peanut are tetraploid and known to have inadequate levels of resistance to several biotic and abiotic constraints. In contrast, the closely related wild relatives of peanut are diploid and possessed several useful disease/pest resistant traits (Holbrook and Stalker, 2003; de Paula et al., 2017). The genetic barrier of “diploid vs tetraploid” prohibited the gene flow from wild-relatives to cultivars and thereby resulted in a narrow genetic base of peanut (Stalker, 2017). In this scenario, allotetraploid and amphidiploid synthetic peanut varieties have been generated which possessed several desirable traits with wide genetic variability of wild germplasm (Mallikarjuna et al., 2011). This provided an opportunity for introgression of resistance traits from eminent synthetic varieties into cultivated peanut gene pool by developing interspecific hybrid varieties (Mallikarjuna et al., 2011; Kumari et al., 2014). Likewise, foliar disease resistance in peanut has been achieved by introgression of traits from synthetic amphidiploids namely ISATGR 278-18 (*A. duranesis* x *A. batizocoi*) and ISATGR 5B (*A. magna* x *A. batizocoi*) into five (ICGV 91114, ICGS 76, ICGV 91278, JL 24, and DH 86) cultivars (Kumari et al., 2014). Also, pest (*Spodoptera litura*) resistant peanut hybrids have been developed by ingression of traits from amphidiploid An13 (*A*. *magna* V 13751 x *A*. *kempff-mercadoi* V 13250) into *A. hypogaea* cv. IAC OL4 (Mallikarjuna et al., 2004; Fávero et al., 2015). In this view, the present study is focused on purification of PIs from interspecific hybrid peanut variety (4368-1) and exploitation of their biophysical and biochemical properties in providing pest resistance against *H. armigera.*

## Materials and Methods

### Seed Material and Insects

Peanut seeds (Interspecific advanced hybrid variety 4368-1) were provided by the International Crop Research Institute for Semi-Arid Tropics (ICRISAT) located in Patancheru, Hyderabad, Telangana, India. The egg mass of *H. armigera* was procured from the National Bureau of Agricultural Insect Resources (NBAIR-MP-NOC-01: *H. armigera*), Bengaluru, India.

### Chemicals

Bovine serum albumin, bovine α-trypsin, α-chymotrypsin, casein and Polyvinylpyrrolidone (PVP) were procured from Sisco Research Laboratory, Mumbai, India. CNBr activated Sepharose 4B, Sephadex G-50 fine, BAPNA, GLUPHEPA, soybean trypsin-CI (soybean BBI), tricine, gelatin, phenylmethylsulfonyl fluoride (PMSF), *N*α-Tosyl-L-lysine chloromethyl ketone hydrochloride (TLCK), *N*p-Tosyl-L-phenylalanine chloromethyl ketone (TPCK), Trizol reagent and Coomassie brilliant blue R-250 were purchased from Sigma Aldrich, United States. Immobiline pH gradient dry strips (IPG strips), IPG buffer, DTT, IDA, urea, thiourea, 3-[(3-Cholamidopropyl) dimethylammonio]-1-Propanesulfonate hydrate (CHAPS), CM5 sensor chips, amine coupling kit and HBS EP + 10X buffer were procured from GE Healthcare Biosciences Corp., United States. Verso cDNA synthesis kit, 50 bp DNA ladder, bicinchoninic acid (BCA) protein estimation kit, protein molecular mass standard, and 3 kDa cut-off SnakeSkin dialysis membrane were purchased from Thermo Fisher Scientific, United States. SYBR Green PCR Master Mix purchased from Takara Bio, Shiga, Japan and all other PCR components from New England Biolabs. Amicon ultra centrifugal filter units were purchased from Millipore Corporation, United States and all other chemicals and reagents used were of analytical grade.

### (NH_4_)_2_SO_4_ Fractionation and Chromatography

Mature dry peanut seeds were blended into a fine powder, pigments and fats were removed by subsequent washes with acetone and hexane. The dried seed powder was extracted against buffer (50 mM Tris-HCl, pH 8.0) with 1% PVP (w/v) by gentle stirring at 4°C for overnight and the resulting supernatant is called as peanut crude proteinase inhibitor (PnCPI) extract (Prasad et al., 2010c).

The clear PnCPI extract was subjected to 0-20%, 20-60%, 60-80% (NH_4_)_2_SO_4_ fractionation. The 20-60% pellet fraction with prominent TI activity was purified by passing successively through trypsin bound CNBr Sepharose 4B column (XK 16/20, 3 cm, 60 ml/h flow rate) and Sephadex G-50 fine column (XK 16/100, 85 cm, 30 ml/h flow rate) by fast protein liquid chromatography (FPLC) system (AKTA prime plus, GE Healthcare). The eluted protein fractions (1.0 ml) from each chromatographic column were analyzed for TI activity. The fractions with significant TI activity were collected and subjected to dialysis (3 kDa c/o) followed by freeze-drying (Labconco, United States)/Amicon filters (3 kDa c/o). The purified protein with prominent TI activity was estimated for protein content and stored at −20°C for further use.

### Electrophoresis

The purity of peanut Bowman-Birk inhibitor (PnBBI) was evaluated by Tricine SDS-PAGE (15%) according to the method of Schägger (2006) under reducing and non-reducing conditions. The various fractions of different purification steps were analyzed electrophoretically along with protein molecular mass standards (4.6 to 100 kDa) and commercial soybean BBI (8.0 kDa) dissolved in 50 mM Tris-HCl, pH 8.0. PnBBI was reduced with 50 mM DTT at 56°C for 1 h followed by alkylation with 100 mM IDA for 45 min in the dark at room temperature before loading on the gel. Native-PAGE of PnBBI was performed using 4% stacking and 12.5% resolving gel by following method of Laemmli (1970). Proteins were identified by staining either CBB R-250 (0.1%) or silver nitrate. Whereas the TI or CI bands were detected in gelatin native/SDS-PAGE followed by CBB staining as described by Felicioli et al. (1997) and Swathi et al. (2014).

### Two-Dimensional Gel Electrophoresis (2-DE)

Isoelectric focusing (IEF, First dimension) was carried out with immobilized pH gradient dry strips pH 4-7, 11 cm (Linear) by Ettan IPGPhor 3 IEF system (GE Healthcare Life Sciences) according to the manufacturer’s guidelines. IPG strip was subjected to passive rehydration at 25°C for overnight with 200 μl of rehydration buffer containing 70 μg of PnBBI under reducing (7.0 M urea, 2.0 M thiourea, 4% CHAPS, 50 mM DTT and 1% IPG buffer) or non-reducing (10% sorbitol and 1% IPG buffer) conditions (Swathi et al., 2014). IEF was carried out at a current setting of 50 μA per strip and applied voltage as follows: 500 V-500 Vh; linear gradient to 1000 V-800 Vh; elevated gradient to 6000 V-8800 Vh and final focusing at 6000 V-4500 Vh. Following the first dimension, each strip was equilibrated for 20 min in equilibration buffer (6 M urea, 30% glycerol and 2% SDS in 0.1 M Tris-HCl, pH 8.8) with 50 mm DTT and 100 mM IDA under reducing or without DTT and IDA under non-reducing conditions. The second dimension was carried out by 15% SDS-PAGE/Gelatin SDS-PAGE.

### MALDI-TOF and MALDI TOF-TOF Analysis of PnBBI

The molecular mass of intact PnBBI was determined by mixing it with a saturated solution of α-cyano-4-hydroxycinnamic acid (CHCA, prepared in 50% ACN and 0.1% TFA in water) and spotting on MALDI metal target plate according to the method followed by Prasad et al. (2010c). MALDI-time of flight (TOF) spectrum was obtained in linear mode by irradiating the air-dried matrix containing PnBBI with laser pulses (Nd: YAG laser) in Bruker Daltonics Autoflex III smartbeam instrument (Bremen, Germany). The isoinhibitor spot of PnBBI (pI 5.9) was excised from a single reduced 2-DE gel (Figure 3A) and subjected to DTT reduction (10 mM) followed by alkylation (55 mM IDA) and digested with trypsin (12.5 μg/μl) before MALDI-TOF-TOF analysis as described in Swathi et al. (2014). The mass spectrometry proteomics data has been deposited to the ProteomeXchange Consortium (Deutsch et al., 2017) via the PRIDE (Perez-Riverol et al., 2019) partner repository with the dataset identifier PXD016933. Biotools (Bruker Daltonics, Version 3.1) was used to analyze the spectrum after MALDI-MS-MS ionization. The peptide sequence obtained from MGF file was subjected to ‘BLASTp’ search in the NCBI database and closely resembled sequences were aligned using the Clustal Omega tool.

### Surface Plasmon Resonance (SPR) and Competitive Binding Assay

Biomolecular interactions between immobilized ligand PnBBI and analyte bovine trypsin or chymotrypsin were determined using the kinetic module of Biacore T200 instrument (GE Healthcare, Life Sciences). The PnBBI (50 μg/ml) was covalently immobilized on the surface of carboxymethyl dextran-5 (CM5) sensor chip by amine coupling principle using 10 mM sodium acetate buffer, pH 4.0. Briefly, the dextran matrix of sensor chip surface (Fc-2) was activated with a mixture of 1-ethyl-3-(3-dimethylaminopropyl)carbodiimide (EDC) followed N-hydroxysuccinimide (NHS) to generate reactive succinimide esters which spontaneously bind to the ligand (PnBBI). The residual active NHS esters on the chip were blocked by passing ethanolamine-HCl after adequate immobilization of PnBBI. The reference flow cell (Fc-1) was treated in the same way without ligand. The kinetic experiment was performed by sequential passing (thrice) of different concentrations (1.56, 3.12, 6.25, 12.5, 25, 50, and 100 nM) of trypsin and/or chymotrypsin dissolved in 0.1 M HEPES buffer containing 1.5 M NaCl, 30 mM EDTA and 0.5% surfactant P20, pH 7.4. Standard conditions (flow rate of 30 μl/min, contact time 180 s and dissociation time 600 s, regeneration contact time 30 s at 25°C) were maintained for each cycle of association, dissociation and regeneration carried out by Glycine HCl, pH 2.0 as described in Biacore manual. A Biomolecular interaction on the sensor chip is represented as a change in Response Unit (RU) in a sensorgram against time in sec. The obtained data analysis was performed using BIA evaluation software (version 4.0, GE Healthcare Life Sciences) with Langmuir fit model of 1:1 binding.

The competitive binding experiment was performed to determine the specificity of two reactive sites of double-headed ligand (PnBBI) toward trypsin and chymotrypsin by following the Biacore T200 control software (capture method module). The flow cell (FC 2-1) of CM5 sensor chip immobilized with PnBBI was confined with saturated (500 nM) levels of first analyte (chymotrypsin/trypsin) followed by passing over (30 μl/min) different concentrations (3.12, 6.25, 12.5, 25, 50, and 100 nM) of second analyte (trypsin/chymotrypsin). The capture process was regenerated by removing both the analytes from PnBBI surface. The disproportion in the number of bound molecules of trypsin or chymotrypsin was interpreted by the difference in RU.

### Assay of Proteases

The protease activity was identified by observing the formation rate of *p-*nitroanilide from BAPNA or GLUPHEPA (1 mM) at 410 nm using UV-visible spectrophotometer (UV-1700, Shimadzu, Japan) as described in Mohanraj et al. (2019). The *p*-nitroanilide molar extinction coefficient (M^–1^ cm^–1^) is corresponding to 8800 at 410 nm. One unit of corresponding trypsin, chymotrypsin, HaTP or HaCP activity is considered as the amount of enzyme or gut extract that raise the reaction absorbance by 1.0 OD after 45 min at 37°C (Figure 7D). Further, the activity of HaTP/HaCP was expressed as moles of *p*-nitroanilide released/min/mg protein as described by Swathi et al. (2014).

### Inhibitory Activity of PnCPI and PnBBI

The inhibitory activity of PnCPI/PnBBI against trypsin, chymotrypsin, HaTP and HaCP was examined indirectly by recording the inhibition of corresponding protease activity. Thus, single unit of TI, CI, *H. armigera* midgut trypsin-like protease inhibitor (HaTPI) or chymotrypsin-like protease inhibitor (HaCPI) activity is considered as the concentration of PnCPI/PnBBI requisite to inhibit 50% of BAPNA or GLUPHEPA hydrolysis by their corresponding proteases under standard assay conditions (Figure 7A). The IC_50_ of PnBBI against trypsin, chymotrypsin or HaTP was identified by monitoring the activity of corresponding proteases followed by incubation with PnBBI at a wide spectrum of concentrations. The inhibitory activity of PnBBI toward different proteases was compared with commercially available soybean BBI (Table 2).

### Stability of PnBBI Against Temperature, pH and Reducing Agent

The influence of temperature on TI or CI activity of PnBBI was examined by incubating it at a broad range of temperatures (20-90°C) for 30 min. Whereas the impact of pH was determined by incubating it in the following buffers at a final concentration of 50 mM: Glycine-HCl (pH 2-3), sodium acetate-acetic acid (pH 4-5), sodium phosphate buffer (pH 6-7), Tris-HCl (pH 8-9) and Glycine-NaOH (pH 10-12) at 37°C. Likewise, the stability of TI or CI activities of PnBBI against reducing agent DTT was assessed by incubating PnBBI with increasing concentration of DTT (0.2, 0.4, 0.6, 0.8, 1.0, and 2.0 mM) followed by alkylation with IDA at two-fold excess of DTT for 1 h in dark. The residual TI or CI activities after incubating at various temperatures, pH and reducing conditions were assayed using BAPNA and GLUPHEPA, respectively, as described in section “Inhibitory Activity of PnCPI and PnBBI.”

### Circular Dichroism Spectroscopy

The secondary structure of PnBBI was determined at far-UV (190-260 nm) range using CD spectroscopy (JASCO J-810, Japan). The spectrum was recorded at 25°C using different PnBBI concentrations (0.2-0.6 mg/ml) in 10 mM PBS under the following conditions: scan speed of 50 nm/min; data pitch and bandwidth of 1 nm; cuvette path length of 2 mm and cuvette volume 0.5 ml of protein solution. Effect of pH and reducing agent on the secondary structure of PnBBI was determined by incubating PnBBI (0.3 mg/ml) in respective pH buffer for 1 h as described above. Similarly, reduction of PnBBI was carried out by treating with 2 mM DTT followed by 4 mM IDA before recording the far-UV spectra. The structural stability of PnBBI at various temperatures was assessed by recording the far-UV CD spectra after every 10°C interval from 20-90°C using the Peltier thermostat. The final spectrum is an average of three scans and the detection chamber including the sample cell was continuously flushed with nitrogen gas throughout the operation. Analysis of CD spectra was performed using DichroWeb online server (Whitmore and Wallace, 2004, 2008). The unit of ellipticity was represented as millidegrees.

### *In vivo* Feeding Bioassay of *H. armigera*

The influence of PnBBI on *H. armigera* larval growth was assessed by performing the *in vivo* feeding assays. The newly hatched larvae on fresh castor leaves were transferred onto the chickpea-based artificial diet (Gupta, 2000; Swathi et al., 2016). Insect culture was maintained at temperature (26 ± 1°C), relative humidity (65 ± 5) and photoperiod (14:10 h) in insect culture room. The *H. armigera* second instar larvae were transferred on to a test diet that is supplemented with low (0.001%) moderate (0.0025%) and high (0.005%) concentrations of PnBBI which is equivalent to 250, 625, and 1250 HaTPI units per gram diet, respectively. For each concentration, 30 numbers of larvae which are approximately equal in size and weight were chosen for the study and maintained in individual cages of insect culture plates. The control larvae were maintained on diet devoid of PnBBI. Observations were recorded periodically by considering the larval body weights, midgut protease activity and changes in trypsin-like and chymotrypsin-like gene expression.

### Extraction of *H. armigera* Midgut Proteases (HaGPs) and Zymography

The midgut from fifth instar larva was dorsally dissected by narcotizing the insects on ice for 30 min using iso-osmotic saline. Proteases were extracted by homogenizing the gut tissue using glass homogenizer in 0.1 M Glycine-NaOH, pH 10.5 followed by centrifugation at 12,000 rpm for 10 min at 4°C. The obtained clear supernatant enriched with HaGPs was used in further *in vitro* enzyme assays and electrophoretic activity staining studies.

The total protease profile of HaGP extract from larvae fed on control or test diet supplemented with different concentrations of PnBBI was visualized by Native-PAGE (4% stacking and 7.5% resolving gel) coupled zymography according to the method of Laemmli (1970) and Prasad et al. (2010a). The type of protease was determined by incubating the HaGPs with various synthetic inhibitors such as PMSF (20 mM) - serine protease inhibitor; TLCK (10 mM) - TI and TPCK (10 mM) - CI at 30°C for 30 min before loading into the gel.

In case of 2-DE, HaGP extract was subjected to acetone precipitation. The pellet obtained was washed thrice with 80% chilled acetone and dissolved in native 2-DE rehydration buffer containing 10% NP40, 5% glycerol and 0.5% IPG buffer. Followed by IEF the IPG strip was equilibrated in 50 mM Tris-HCl, pH 8.8 containing 2% SDS, 30% glycerol for 25 min (as described in section ‘2-DE’). The second dimension was carried out on 10% SDS-PAGE followed by zymography. After electrophoresis excess detergent was removed by washing the gels thrice with 2.5% Triton X-100 followed by distilled water. Subsequently, gels were incubated in chilled buffer (0.1 M Glycine-NaOH, pH 10.5) containing 1% casein for 30 min under mild shaking. The casein solution was drained after incubation at 37°C for 1 h, washed with distilled water and protease activity bands were visualized as clear zones where substrate casein was hydrolyzed by proteases in the gel after staining with CBB R-250. Finally, differential protease activity profiles were compared between larvae fed on the control diet and high dose PnBBI diet.

### RNA Isolation and Quantitative Real-Time PCR (qRT-PCR)

The midgut tissue (pool of six guts) free from ingested food material was collected at time intervals of day3, 6, and 9 from *H. armigera* larvae fed on diet in presence and absence of PnBBI as described above and added to 0.5 ml of TRI reagent (Sigma-Aldrich, St Louis, MO, United States) for isolation of RNA. The tissue was homogenized using micro pestle at ice-cold conditions and centrifuged at 13,000 rpm. The supernatant was mixed with 200 μl of chilled chloroform, vortexed and centrifuged at 13,000 rpm. To the upper layer collected, an equal volume of isopropanol was added to precipitate RNA. Finally, the RNA pellet was washed thrice with 75% ethanol, air-dried and suspended in RNase free water. The quantity and integrity of RNA was determined using Nanodrop (Thermo Scientific, United States) and agarose gel electrophoresis, respectively.

The first strand of DNA was synthesized by adding 1 μg of total RNA using Verso cDNA synthesis kit as per manufacturer’s instructions. The obtained cDNA was subjected to 1:40 dilution and selected midgut trypsin-like (*HaTry* 1, 4, 6, 7, 8) and chymotrypsin-like (*HaChy* 1, 2, 4) genes of *H. armigera* were amplified using their respective primers (Supplementary Table 1) and analyzed by agarose gel electrophoresis (Chougule et al., 2005; Chikate et al., 2013; Mahajan et al., 2013). The 40S ribosomal protein gene (RpS18) was used as a reference for normalization. The relative transcript abundance of selected genes was determined by qRT-PCR using the StepOnePlus Real-Time PCR system (Applied Biosystems, United States) and ROX as reference fluorescent dye. The qRT-PCR (10 μl) reaction mixture contains 1.0 μl of cDNA, 5.0 μl of 2X SYBR Green PCR Master Mix, 0.5 μl of each forward and reverse primers and 3.0 μl of sterilized water. PCR conditions were maintained at 94°C for 10 min (initial denaturation), 40 cycles of 94°C for 15 s followed by 60°C for 1 min for annealing and extension. The respective changes of gene expression were assessed by 2^–ΔΔ*CT*^ method using the cycle threshold (C_*T*_) value of qRT-PCR analysis (Livak and Schmittgen, 2001). The C_*T*_ value of genes of interest (GOI) were normalized against C_*T*_ value of a housekeeping gene (RpS18) and ΔC_*T*_ value (ΔC_*T*_ = GOIC_*T*_ – RpS18C_*T*_) was generated. Later, ΔΔC_*T*_ (ΔΔC_*T*_ = TestΔC_*T*_ – ControlΔC_*T*_) value generated is finally represented in log2 fold change of 2^–ΔΔ*CT*^.

### Statistical Analysis

The data shown is Mean ± SE/SD of three biological replicates. Statistical differences were determined by one-way ANOVA followed by Tukey test at a significance level of *P* ≤ 0.05 using Sigma plot, version 12.0, Systat Software Inc., San Jose, CA, United States. Biological replicates for relevant figures are shown in Supplementary Data Sheet 3.

## Results

### Purification of Peanut Proteinase Inhibitor (PnBBI)

**FIGURE 1:**
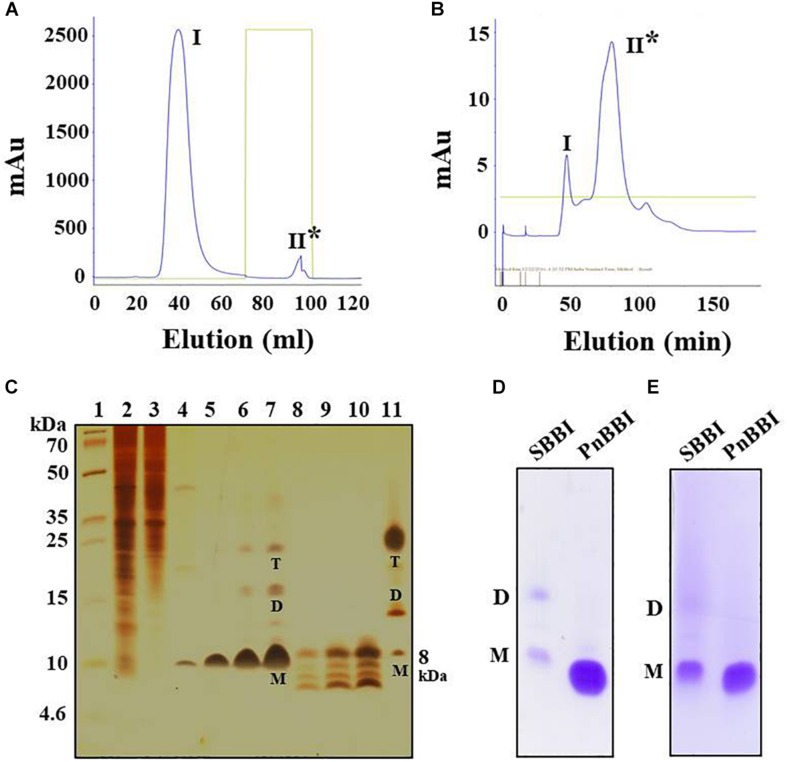
Purification profile of PnBBI and its in-gel activity. Elution profile of **(A)** trypsin-Sepharose 4B column loaded with 20-60% (NH_4_)_2_SO_4_ active fraction; **(B)** Sephadex G-50 fine column loaded with active peak II fraction pool of trypsin affinity column; **(C)** Tricine SDS-PAGE (15%) showing purification profile and self-association pattern of PnBBI: lane 1, molecular weight marker designated in kDa; lane 2, peanut crude protein extract (20 μg); lane 3, 20-60% (NH_4_)_2_SO_4_ protein fraction (15 μg); lane 4, active fraction pool (Peak II) of trypsin affinity column (10 μg); lanes 5-7 and 8-10 active fraction pool (peak II) of gel filtration column under non-reducing and reducing conditions with increased protein concentration (2.5, 5, and 10 μg), respectively; lane 11, soybean BBI (5 μg) was used as a reference. Gelatin SDS-PAGE (15%): Lane 1, soybean BBI (5 μg); lane 2, PnBBI (5 μg) active against **(D)** bovine pancreatic trypsin and **(E)** chymotrypsin, respectively. Asterisks indicate active peak with inhibitory activity against trypsin. M-monomer, D-dimer and T-tetramer. The data shown here is the representative of three biological replicates (data provided as Supplementary Data Sheet 3).

The 20-60% (NH_4_)_2_SO_4_ fraction of PnCPI extract that showed prominent TI activity was subjected to Sepharose 4B trypsin affinity chromatography. The trypsin bound protein (peak II) was eluted using 0.01 N HCl followed by neutralization with Tris base (Figure 1A). The eluted fractions with TI activity were pooled and loaded onto the Sephadex gel permeation column. Among the obtained peaks, protein fractions (peak II) showed prominent TI activity were pooled and concentrated (Figure 1B). The progressive increase in the purity of protein during the sequential steps of purification was clearly evident in SDS-PAGE. Also, an increase in the number of protein bands (monomer, dimer, and tetramer) was observed with an increase in loading concentration of purified protein from 2.5-10 μg (Figure 1C, **lanes 5-7**). The present protocol yielded pure protein with 167-fold purification and 37.5% yield recovery (Table 1). Further, non-reducing gelatin SDS-PAGE in-gel activity staining studies of purified protein demonstrated the presence of TI and CI activities similar to commercial soybean BBI (Figures 1D,E). Hence, it is named as “PnBBI.”

**TABLE 1:** Purification of PnBBI from mature seeds (10 g) of peanut interspecific advanced variety (4368-1).

Purification step	Total protein (mg)	Total activity (TIU)^a^	Yield recovery (%)	Specific activity (TIU/mg)^b^	Purification fold change
Crude extract	713	6000	100	8.4	1
(NH_4_)_2_SO_4_ Fractionation (20-60%)	490	5164	86	10.5	1.3
Trypsin-Sepharose 4B	8.4	2500	41.6	298	35.5
Sephadex G-50	1.6	2250	37.5	1406	167

The data shown here is a representative of the purification protocol for PnBBI from three independent biological replicates*. ^a^ One TI unit is equal to 40-60% inhibition of trypsin enzyme activity by PnBBI. ^b^Specific inhibitory activity is defined as the number of TIU per milligram of PnBBI.*data provided as Supplementary Data Sheet 3.

### Determination of Molecular Mass and Self-Association Pattern

**FIGURE 2:**
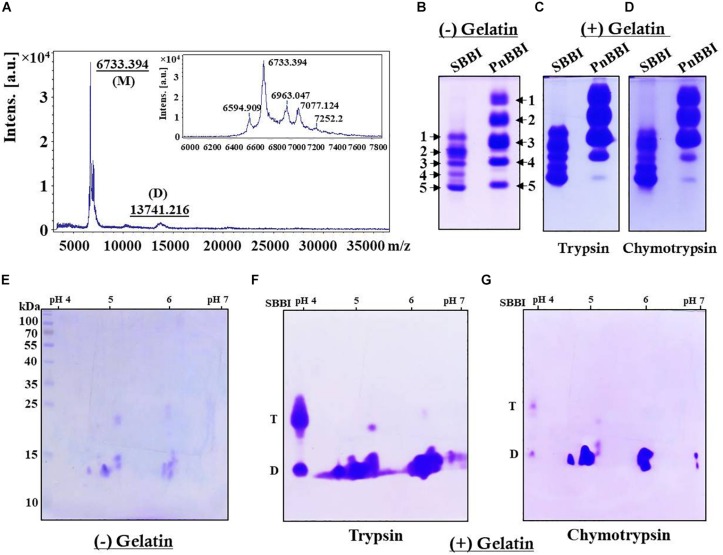
Molecular mass determination and electrophoretic visualization of PnBBI isoinhibitors, and in-gel activity staining. **(A)** MALDI-TOF intact mass spectrum of PnBBI from 3000-37000 *m/z* representing a sharp and high-intensity peak (monomer) with a molecular mass of 6733.394 Da, and its self-associated dimer (13741.216 Da). Inset: zoomed spectrum at 6000-7800 *m/z* represents the peanut isoinhibitors; **(B)** Visualization of PnBBI isoinhibitors (20 μg) resolved on native gel electrophoresis (12.5%). In-gel activity staining against bovine **(C)** trypsin and **(D)** chymotrypsin resolved on gelatin Native-PAGE; **(E)** 2-D electrophoretic separation of PnBBI (70 μg) isoinhibitors (IEF, pH 4-7, 11 cm, L) under non-reducing conditions and respective in-gel activity staining against bovine **(F)** trypsin and **(G)** chymotrypsin. The second dimension was performed on SDS-PAGE (15%) under non-reducing conditions. Gels were stained with CBB R-250. Soybean BBI (SBBI) is loaded as a positive control in Native-PAGE as well as activity staining studies. D-dimer and T-tetramer. The mass spectrum and gel pictures shown here are the selective representatives of two or three biological replicates (data provided as Supplementary Data Sheet 3).

Electrophoretic separation of PnBBI resolved as a single protein with a molecular mass of ∼10 kDa on Tricine SDS-PAGE under non-reducing conditions (Figure 1C, **lane 5**). However, intact mass analysis of PnBBI by MALDI-TOF revealed a predominant peak of 6733.394 Da, as a monomer and a minor peak of 13741.216 Da, as a dimer (Figure 2A). PnBBI existed as several isoinhibitors apparently with similar molecular mass and each isoinhibitor showed variability in their abundance (inset of Figure 2A), which was also evident by Native-PAGE and 2-D electrophoresis (Figures 2B,E). Nevertheless, all the electrophoretically resolved isoinhibitors exhibited resistance against bovine trypsin and chymotrypsin digestion (Figures 2C,D,F,G). The observed self-association tendency and presence of TI and CI activities of PnBBI are in agreement with the characteristic features of PIs belonging to BBI family.

On the other hand, appearance of several new monomeric bands (∼6-10 kDa) was observed in Tricine SDS-PAGE under reducing conditions, parallel to the disappearance of dimeric and tetrameric bands which further confirms the existence of PnBBI in several isoforms as well as higher-ordered oligomeric forms (Figure 1C, **lanes 8-10**). Further, soybean BBI (8 kDa) was used as a reference to eliminate the discrepancy in molecular mass and electrophoretic migration of self-associated BBIs (Figure 1C, **lane 11;** Swathi et al., 2014). However, the molecular mass of PnBBI was considered as “6.73 kDa” as evident in MALDI-TOF studies.

### Two-Dimensional Gel Electrophoresis and MALDI-TOF/TOF Analysis

**FIGURE 3:**
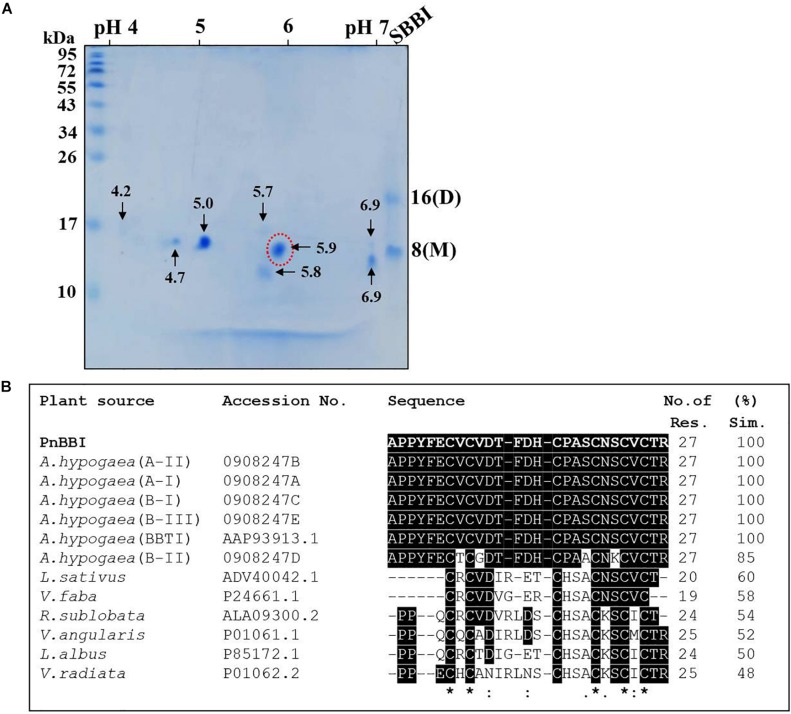
2-D electrophoresis and MALDI TOF-TOF analysis of PnBBI. **(A)** Isoelectric focusing (pH 4-7, 11 cm, L) of PnBBI (70 μg) was performed under standard reducing conditions as described in methods (Section “Two-Dimensional Gel Electrophoresis (2-DE)”). The second dimension was performed on SDS-PAGE (15%) under reducing conditions and the gel was stained with CBB R-250. Molecular mass standards and soybean BBI were used as a reference and positive control, respectively. The 2-DE gel picture shown is the representative of three biological replicates; **(B)** The isoinhibitor spot with pI 5.9 (indicated by a dotted circle of Figure 3A) was subjected to trypsin digestion followed by MALDI TOF-TOF analysis. The resulting peptide sequence from Mascot MS/MS ions search was aligned with the homologous BBI sequences of NCBI database using Clustal Omega tool. The obtained sequence has shown 100% identity with peanut BBI. The gel picture shown here is the selective representation of three biological replicates (data provided as Supplementary Data Sheet 3).

Two-dimensional electrophoretic separation of PnBBI under non-reducing conditions resolved as several isoinhibitor and its higher-order forms in pH range between 4-7 (Figure 2E). Further, gelatin in-gel activity staining studies demonstrated the resistance of these isoinhibitor spots to hydrolysis against both trypsin and chymotrypsin (Figures 2F,G). The high-intensity isoinhibitor spot at pI 5.9 resolved under reducing conditions in 2-DE was excised and subjected to MALDI MS-MS analysis (Figure 3A). The MS-MS ionization of PMF peak 3313.817 m/z obtained the following amino acid sequence “APPYFECVCVDTFDHCPASCNSCVCTR” matched to *Arachis hypogaea* BBI A-II from Mascot MS-MS ions search with a score of 20 and 38% sequence coverage (Supplementary Data Sheets 1, 2). Also, the lift spectrum generated using Biotools matched with the above sequence (Supplementary Figure 1). Further, Blastp search of the obtained sequence showed 100% identity with BBIs from *A. hypogaea* (A-I, B-III, BBTI and B-II) and 48-60% similarity with other legume BBIs such as *Lathyrus sativus*, *Vicia faba*, *Rhynchosia sublobata*, *Vigna angularis*, *Lupinus albus* and *V. radiata*. Multiple sequence alignment of the obtained sequence is in accordance with the pattern of conserved cysteine residue framework of BBIs (Figure 3B).

### Kinetic Analysis Using SPR

**FIGURE 4:**
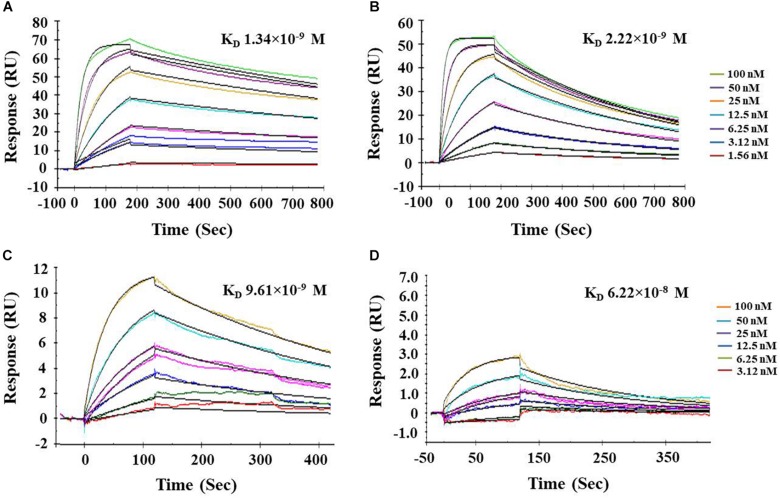
Biacore surface plasmon resonance (SPR) analysis of PnBBI. Sensogram plots generated by SPR kinetic analysis demonstrate the association and dissociation characteristics between immobilized ligand (PnBBI) and analytes, bovine **(A)** trypsin and **(B)** chymotrypsin; Competitive binding studies between ligand and analytes where **(C)** Chymotrypsin (500 nM) was captured on PnBBI ligand and trypsin was passed through as an analyte and **(D)** Trypsin (500 nM) was captured on PnBBI ligand and chymotrypsin was passed over as an analyte. A range of trypsin/chymotrypsin concentrations (1.56, 3.12, 6.25, 12.5, 25, 50, and 100 nM) prepared in 10 mM HEPES, pH 7.4 are passed over the surface of PnBBI sequentially and the curves obtained were further analyzed by Langmuir fit model of 1:1 binding. One response unit (RU) corresponds to a change of 1 pg/mm^2^ in surface protein concentration i.e., analytes. The final sensorgram is representative of three cycles as described in Section“Surface Plasmon Resonance (SPR) and Competitive Binding Assay.”

Surface plasmon resonance analysis has been widely used to study the biomolecular interactions in the real-time and label-free environment. The ligand PnBBI showed stable interactions with both trypsin (Ka = 4.18 × 10^5^ M^–1^s^–1^; Kd = 0.00056 s^–1^) and chymotrypsin (Ka = 7.76 × 10^5^ M^–1^s^–1^; Kd = 0.00172 s^–1^) during SPR kinetic analysis. The obtained dissociation equilibrium constant emphasize the relative stability of PnBBI-trypsin (K_*D*_ = 1.34 × 10^–9^ M) complex over PnBBI-chymotrypsin (K_*D*_ = 2.22 × 10^–9^ M) complex with slower dissociation rates (Figures 4A,B). On the other hand, quantitative binding analysis of trypsin and chymotrypsin to double-headed PnBBI was determined by SPR competitive binding assay (Figures 4C,D). These results suggest the chymotrypsin saturated PnBBI surface showed greater capacity to bind with trypsin molecules which is evident by maximum RU of 12.6. In contrast, trypsin saturated PnBBI surface showed a lower capacity to bind with chymotrypsin molecules which is evident by maximum RU of 2.46.

### Functional Stability

**FIGURE 5:**
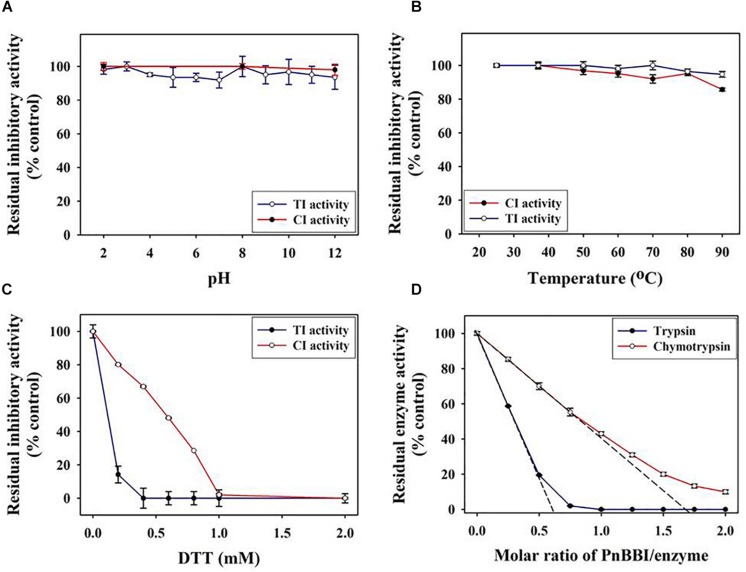
Functional stability of PnBBI. The TI and CI activities of PnBBI were monitored at a broad range of **(A)** pH; **(B)** temperature and **(C)** reducing agent Dithiothreitol (DTT). The amount of PnBBI equivalent to one TI or CI units was incubated at different pH, temperatures or DTT concentration and residual inhibitory activities against trypsin and chymotrypsin was monitored using respective substrates as described in methods (Section “Inhibitory Activity of PnCPI and PnBBI”); **(D)** Titration curve of trypsin and chymotrypsin inhibition by PnBBI. A fixed amount of enzyme (1 μM trypsin and/or 2 μM chymotrypsin) was mixed with different concentrations of PnBBI and the residual protease activity was determined. The x-intercept where the corresponding proteases are completely inhibited by the inhibitor was the molar binding ratio. The data represented is mean ± SE of three biological replicates.

The TI and CI activities of PnBBI was stable even after incubating at a wide range of pH (2-12) and temperature (20-90°C). PnBBI lost < 5% of TI activity and < 14% of CI activity under the above extreme conditions (Figures 5A,B). In contrast, the TI, CI activities of PnBBI were completely (100%) lost upon incubation with an increasing concentration of reducing agent (DTT) followed by alkylation (IDA). The TI activity of PnBBI was found to be much sensitive to DTT reduction compared to CI activity in which 14% of TI and 80% of CI activity was retained at 0.2 mM DTT (Figure 5C). This result emphasizes the vital role of disulfide bonds in stabilizing the structure of trypsin reactive loop of PnBBI. The superior affinity of PnBBI toward trypsin was evident by molar inhibition studies, in which PnBBI binds with trypsin at 1:2 stoichiometry but not with chymotrypsin (Figure 5D).

### Structural Stability

**FIGURE 6:**
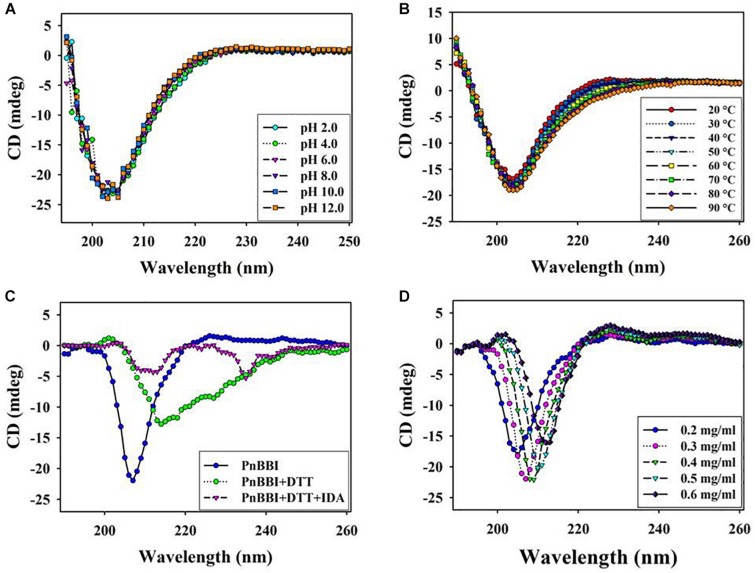
Structural stability of PnBBI. Far-UV (190-260 nm) secondary structure of PnBBI was determined by CD spectroscopy at different **(A)** pH from 2.0 to 12.0; **(B)** temperatures from 20 to 90°C; **(C)** DTT (2 mM) reduction followed by IDA (4 mM) alkylation and **(D)** increasing concentration (0.2 - 0.6 mg/ml) of PnBBI prepared in 10 mM PBS, pH 7.4 as described in methods. The final spectrum is an average of three scans as described in Section “Circular Dichroism Spectroscopy.”

Circular dichroism spectroscopy is the most useful technique for studying protein secondary structure in solution. The DichroWeb analysis of far-UV CD spectrum revealed the predominance of β-sheets (47%) and random coils (44%) over α-helix (9%) in PnBBI. The secondary structure of PnBBI has not shown much variation when incubated under different pH or temperatures (Figures 6A,B). However, a significant distortion in the secondary structure of PnBBI was observed upon reduction and alkylation with DTT and IDA, respectively (Figure 6C). In contrast, there was a shift in the negative ellipticity maxima of spectrum toward higher wavelength i.e., from 205 - 212 nm with an increase in PnBBI concentration from 0.2 to 0.6 mg/ml (Figure 6D).

### Effect on *H. armigera* Larval Growth

**FIGURE 7:**
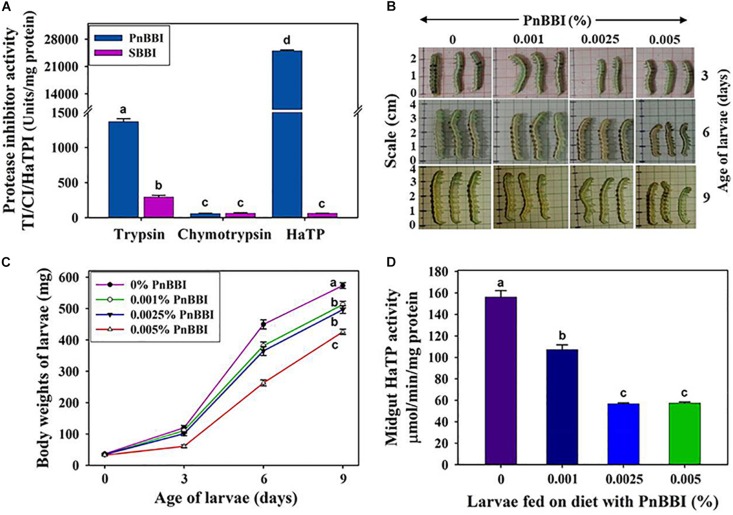
*In vitro* and *in vivo* inhibitory effect of PnBBI on larvae of *H. armigera*. **(A)** Comparative inhibitory profiles of PnBBI and SBBI toward bovine trypsin, chymotrypsin and HaTP; **(B)** Photographs depicting the reduction in size of larvae fed on increasing doses (0.001, 0.0025, and 0.005%) of PnBBI containing diet as compared to larvae fed on control diet at the end of 3, 6 and 9 days of feeding. A set of thirty number of second instar larvae with approximate equal body size and weight were used in the present study for feeding with each concentration of PnBBI; **(C)** depletion in mean body weights of larvae after feeding on diet containing PnBBI as described above; **(D)** variation in the activity of midgut HaTPs in larvae after feeding on diet containing PnBBI as described above. The data represented is mean ± SE of three biological replicates. Different lower case letters “a, b, c, and d” indicate statistically different at a significant level (*P* < 0.05) compared to control.

The inhibitory potential of PnBBI against *H. armigera* larval growth was examined under both *in vitro* and *in vivo* conditions. Under *in vitro* conditions, PnBBI exhibited strong inhibitory activity against trypsin-like HaGPs (25,000 HaTPI units/mg protein) as compared to bovine trypsin (1406 TI units/mg protein) and chymotrypsin (57 CI units/mg protein), respectively (Figure 7A). Also, it has strong inhibitory potential against HaTP (IC_50_ = 40 ng) when compared with soybean BBI (IC_50_ = 16.5 μg). In contrast, PnBBI has not shown much variation with soybean BBI in its inhibitory potential against chymotrypsin (Table 2). Further, *in vivo* feeding experiments demonstrated a dose-dependent reduction in larval body mass of *H. armigera* upon feeding with increasing concentrations (0.001, 0.0025 and 0.005%) of PnBBI (Figures 7B,C). Among the given treatments, maximum reduction (42% of control) in body mass of larvae was observed on day 6 when fed upon high dose (0.005%) of PnBBI (Figure 7C). The antifeedant potential of PnBBI was further evaluated by comparing the activity of serine proteases in the midgut of larvae fed on various doses of PnBBI using *in vitro* studies. A gradual decrease (∼63%) in the activity of HaTPs was observed in larvae fed upon the moderate and high dose of PnBBI as compared to larvae fed on control diet (Figure 7D). However, the chymotrypsin-like activity of HaGPs was not in detectable range in control as well as treated larval gut extracts.

**TABLE 2:** Comparison of inhibitory potential of PnBBI and soybean BBI toward Bovine trypsin, chymotrypsin and trypsin-like midgut proteases of *H. armigera* (HaTPs).

Protease	Half-maximal inhibitory concentration (IC_50_)	
	PnBBI (μg)	Soybean BBI (μg)
Bovine trypsin	0.730 ± 0.01	3.4 ± 0.09
Chymotrypsin	17.54 ± 0.92	16.1 ± 0.85
HaTPs	0.040 ± 0.003	16.5 ± 0.65

*The data represented is mean ± SE of three biological replicates. IC_50_ is defined as the amount of inhibitor required to inhibit half of the corresponding protease activity.*

### In-gel HaGP Activity Profile and Their Inhibition

**FIGURE 8:**
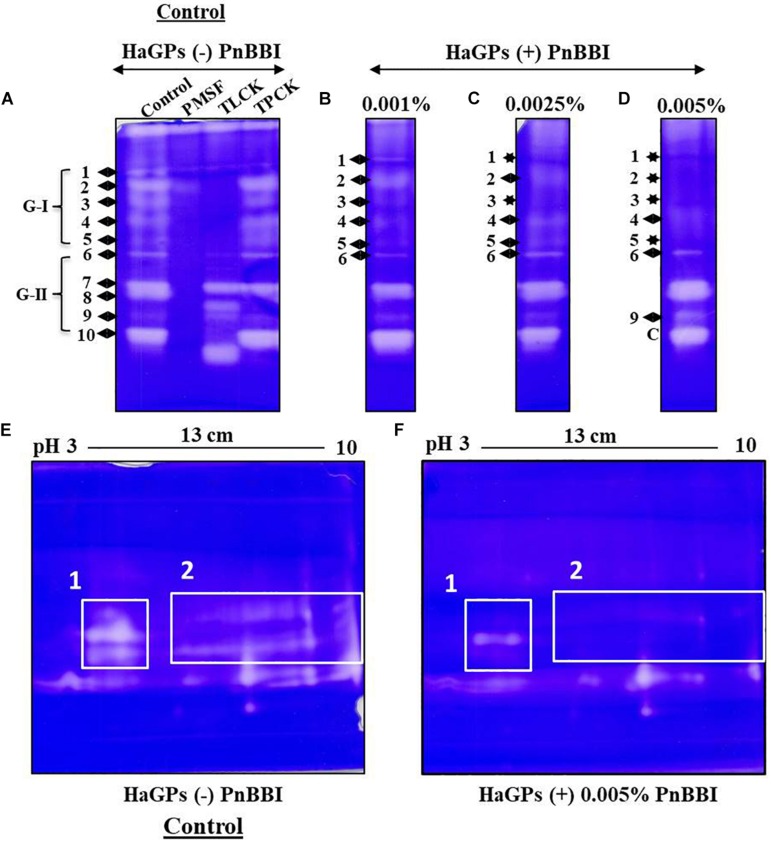
Comparative Zymography of HaGPs from larvae fed on PnBBI. Casein Native-PAGE zymogram of HaGPs extracted from larvae (day9) fed on diet with **(A)** No PnBBI (Lane1: HaGPs, lane2: HaGPs + PMSF, lane3: HaGPs + TLCK and lane4: HaGPs + TPCK); **(B)** 0.001% PnBBI; **(C)** 0.0025% PnBBI and **(D)** 0.005% PnBBI. Differential 2-DE zymography of HaGPs from larvae fed on **(E)** control diet and **(F)** 0.005% PnBBI supplemented diet. An equal amount of protein (8 μg) from both samples was subjected to rehydration of IPG strip under native condition followed by IEF, second dimensional SDS-PAGE (10%) and zymography as described in materials and methods (Section “Extraction of *H. armigera* Midgut Proteases (HaGPs) and Zymography”). The respective clear zones against the dark blue background are the result of casein digestion due to the presence of proteases. The area highlighted with boxes on 2-DE zymograms represents the disappearance of several proteases upon PnBBI ingestion as indicated against stars. The gel pictures are the selective representatives of two biological replicates (data provided as Supplementary Data Sheet 3).

The activity of HaGP(s) in larvae fed on control diet was detected as 10-12 distinct protease bands on casein Native-PAGE. The disappearance of this protease band pattern upon incubation with PMSF demonstrated the predominance of serine proteases in midguts of *H. armigera* larvae. However, the protease activity bands 1-5 (group-I) were inhibited by trypsin-like protease inhibitor TLCK, but not by chymotrypsin-like protease inhibitor TPCK. Also, protease band 9 disappeared in the presence of TPCK but not in presence of TLCK. These results suggest that group-I bands belong to trypsin-like midgut proteases and band 9 belong to chymotrypsin-like protease in HaGP extract (Figure 8A). Mimicking the treatment with TLCK, the activity of HaTPs (bands 1-5 of HaGPs) was decreased gradually with increasing concentration of PnBBI in the diet supplemented to larvae. Apart, at high dose (0.005%) of PnBBI, the activity of protease band 9 (HaCP) increased when compared with the control sample (Figures 8B-D). A great divergence was observed in protease profiles of larvae fed on high dose of PnBBI when compared with the control. It was further resolved using 2-DE coupled zymography technique. HaGPs of larvae fed on the control diet showed a greater number of active protease spots ranging between pH 3 to 10. In contrast, several of these protease spots disappeared in zymogram of HaGPs extracted from larvae fed on a high dose (0.005%) of PnBBI as indicated in box 1 and 2 (Figures 8E and F).

### qRT-PCR Analysis of HaGPs

**FIGURE 9:**
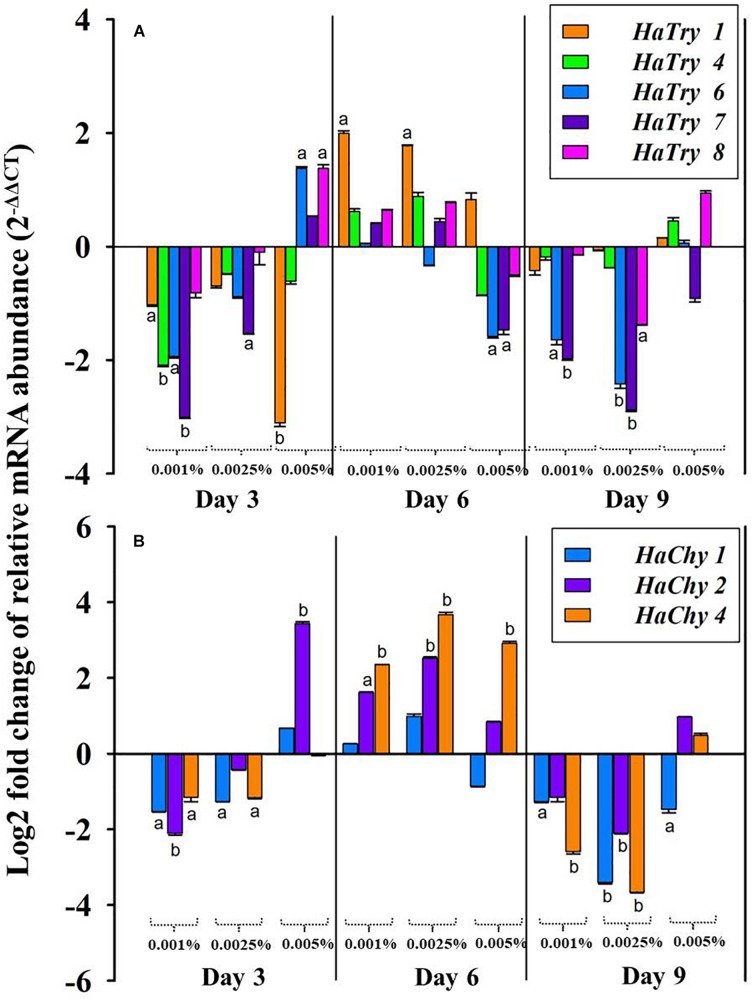
qRT-PCR analysis of midgut serine proteases of *H. armigera* larvae fed on PnBBI. Changes in transcript abundance of **(A)** midgut trypsin-like (*HaTry* 1, 4, 6, 7, and 8) and **(B)** chymotrypsin-like (*HaChy* 1, 2, and 4) proteases from midgut tissue of *H. armigera* larvae after rearing on artificial diet containing different concentration of PnBBIs (0, 0.001, 0.0025, and 0.005%) for 3, 6, and 9 days. The fold difference in transcript abundance of these candidate genes was calculated using Livak (2^– ΔΔ*CT*^) method and final values are expressed in log (base2) fold changes. Further details were as described in the methods section “RNA Isolation and Quantitative Real-Time PCR (qRT-PCR).” The data shown is mean ± SD of triplicates. The transcript abundance of trypsin and chymotrypsin-like genes for the larvae fed on PnBBI incorporated diet was compared with control diet-fed larvae in which lower case letters “a and b” assigned for transcripts which showed > 1 and 2-fold difference in their abundance, respectively.

The quantitative real-time PCR analysis revealed the relative transcript abundance of HaTPs (*HaTry*1, *HaTry*4, *HaTry*6, *HaTry*7, *HaTry*8) and HaCPs (*HaChy*1, *HaChy*2, *HaChy*4) in larvae fed on low (0.001%), moderate (0.0025%) and high (0.005%) dose of PnBBI at the end of day3, day6 and day9 (Figure 9). The five HaTP-like (*HaTry* 1, 4, 6, 7, and 8) and two HaCP-like (*HaChy* 2 and 4) genes were selected from the study of Chikate et al. (2013) and Mahajan et al. (2013) and the remaining *HaChy* 1 was adopted from Chougule et al. (2005). At the end of day3 and day9, relative transcript abundance of HaTPs was down-regulated (< 3-fold) at low as well as at a moderate dose of PnBBI, while most of the HaTPs (except *HaTry*1/*HaTry*4/*HaTry*7) were up-regulated (< 2-fold) at a high dose of PnBBI. In contrary, at the end of day6, the relative transcript abundance of HaTPs was increased (< 2-fold) at a low or moderate dose of PnBBI, while most of the HaTPs (except *HaTry*1) were down-regulated (< 2-fold) at a high dose of PnBBI (Figure 9A).

The relative transcript abundance of HaCPs were down-regulated (< 3.7-fold) similar to HaTPs at the end of day3 and day9 in presence of low or moderate dose of PnBBI. However, the transcript levels *HaChy*2 was up-regulated significantly at the end of day3 (< 3.7-fold) in the presence of a high dose of PnBBI. In contrast, at the end of day6, the transcript levels of all HaCPs (except *HaChy*1 at high dose) were up-regulated (< 4-fold) under low, moderate and high dose of PnBBI (Figure 9B).

## Discussion

Peanut seeds are rich in protein, edible fats and various bioactive molecules such as PIs (Norioka et al., 1982; Arya et al., 2016). PIs are proteinaceous enzyme inhibitors isolated from several legumes and explored for many applications including therapeutic as well as insecticidal agents (Srikanth and Chen, 2016; Clemente et al., 2019). Though the presence of various BBI isoforms and its crystal structure has been elucidated in some cultivars of peanut, its identification or characterization in interspecific hybrid peanut varieties which are known to possess biotic resistant traits was not yet explored (Suzuki et al., 1987; Mallikarjuna et al., 2011).

### Biochemical and Biophysical Characterization of Macromolecule PnBBI

The PnBBI was isolated using salting-out precipitation and purified to homogeneity using trypsin affinity and size exclusion chromatography techniques (Figures 1A,B). The purity and yield recovery of PnBBI obtained in the present study is much superior when compared with PIs isolated from other plant sources such as mung bean (Klomklao et al., 2011), fenugreek (Oddepally et al., 2013), sombreiro (Dantzger et al., 2015) and pigeon pea cultivars (Swathi et al., 2014) as well as wild relatives (Swathi et al., 2016; Mohanraj et al., 2019) (Table 1). The most discrepancy observed in molecular mass of PnBBI determined by Tricine SDS-PAGE (∼10 kDa) as well as MALDI-TOF (6.73 kDa) analysis is not uncommon among the BBIs (Figures 1C, lane 5 and 2A; Swathi et al., 2014; Mohanraj et al., 2019). However, the observed molecular mass of PnBBI in SDS-PAGE is comparable to commercial soybean BBI. The retarded electrophoretic migration of PIs could be due to their compact structural makeup (Gennis and Cantor, 1976; Bergeron and Nielsen, 1993; He et al., 2017). Though the molecular mass of PnBBI (6.73 kDa) varied marginally, its biochemical properties such as structural and functional stability corroborated well with soybean BBI (Mello et al., 2003; Gu et al., 2014). Further, the amino acid sequence identity of isoinhibitor spot (pI 5.9) with known plant BBIs during MALDI MS-MS analysis confirmed the purified protein belongs to BBI family (Figures 3A,B). Also, peanut BBI resembled with other known plant PIs in its crystal structure and biochemical properties except for non-identical reactive site loops and their specificity toward proteases being inhibited (Figure 3B; Suzuki et al., 1987; Qi et al., 2005). Further, the characteristic features such as low molecular mass and binding specificity of PnBBI is in line with several isoinhibitors of *V. angularis* (Ishikawa et al., 1985), *V. faba* (Gupta et al., 2000) and *L. sativus* (Rocco et al., 2011). Such BBIs identified earlier in pigeonpea and black gram through MALDI-TOF-TOF studies (Prasad et al., 2010b,c; Swathi et al., 2014).

In the present study, concentration-dependent self-association of PnBBI was visible in Tricine SDS-PAGE (Figure 1C, **lanes 5-7**). However, the higher-ordered structures were dissociated after reduction with DTT as monomers (6-10 kDa), suggesting the key role of disulfide bonds in self-association (Figure 1C, **lanes 8-10**). Previous studies indicated that the oligomeric structures of BBIs are known to be stabilized by hydrogen bonds among exposed hydrophobic patches of electrically charged clusters formed due to disulfide bonds (Silva et al., 2005; Rao and Suresh, 2007; Joshi et al., 2013). In case of horse gram, dimers shown to be stabilized by the electrostatic interaction between Lys^24^ and Asp^75/76^ residues of *N*, *C*-terminal ends (Kumar et al., 2004; Muricken and Gowda, 2010). However, the mechanism of self-association may vary among various BBI molecules. The distinctive self-association characteristic feature of PnBBI was further validated by concentration-dependent far-VU CD spectra (Figure 6D). The observed “red shift” from 205 to 212 nm in negative CD band together with decreased intensity was attributed to “absorption flattening” phenomena (Wallace and Teeters, 1987; Manzo et al., 2015). The systematic arrangement of chromophores (oligomer) and their random distribution in the sample perhaps might give differential absorption by a stepwise increase in the concentration of PnBBI. Similar changes in CD spectra associated with protein aggregation were reported earlier even with other proteins such as seed globulins (Mäkinen et al., 2016), antimicrobial peptides (Gopal et al., 2012) and monoclonal antibodies (Joshi et al., 2014). However, the formation of dimer or further oligomers does not induce any steric hindrance while reacting with trypsin which is also evident in 2-DE in-gel activity staining study (Figures 2F,G). Besides, the self-association of BBIs not only provides stability, but also important in molecular packing as a seed storage protein (Hogg, 2003; Kumar et al., 2015). All the isoinhibitors visualized on one and two-dimensional gel electrophoresis conferred resistance against trypsin and chymotrypsin digestion (Figures 2C,D,F,G; Prasad et al., 2010a; Swathi et al., 2016; Mohanraj et al., 2019). The prominent TI activity was evidenced through wider protective zones against trypsin as compared to chymotrypsin during gelatin in-gel activity staining (Figure 2F). However, the existence of BBI as several isoinhibitors is a part of plant defensive trait to combat against pests and their counter defense (Giri et al., 2003; Lopes et al., 2004; War et al., 2018).

The greater inhibitory potential of PnBBI toward trypsin over chymotrypsin is also evident through stoichiometric assays as well as SPR binding analysis (Figures 4C,D, 5D). The TI activity of PnBBI was > 24-fold higher as compared to its CI activity (Table 2). However, the differential inhibitory potential of double-headed PnBBI was attributed to its reactive site amino acids and their specificity toward cognate proteases. Further, the stoichiometric molar ratio of PnBBI and trypsin was found to be 1:2 which suggests both reactive sites can interact with trypsin in the absence of chymotrypsin. In contrast, chymotrypsin has not shown such distinctive stoichiometry, though it was inhibited by PnBBI (Figure 5D; Prasad et al., 2010c; Satheesh and Murugan, 2012). These results taken together suggest that one of the reactive sites of PnBBI is flexible to bind either trypsin or chymotrypsin. Similar binding ratio (1:2) was reported earlier in case of TIs isolated from *L. albus* (Scarafoni et al., 2008), *Vigna mungo* (Prasad et al., 2010b), *Artocarpus heterophyllus* (Lyu et al., 2015) and *Psophocarpus tetragonolobus* (Banerjee et al., 2017).

Further, SPR kinetics suggests PnBBI forms more stable complex with trypsin K_*D*_ = 1.34 × 10^–9^ M compared to chymotrypsin K_*D*_ = 2.22 × 10^–9^ M (Figures 4A,B). In this study, the PnBBI-chymotrypsin complex is able to bind with 5-fold excess (12.6 RU) of trypsin but not reciprocated by PnBBI-trypsin complex (2.46 RU) (Figures 4C,D). These results corroborate well with the reports of Norioka et al. (1982), in which peanut BBI-chymotrypsin complex prone to bind with trypsin by slow release of its partner chymotrypsin but not seen in case of BBI-trypsin complex. PnBBI also possessed excessive anti-parallel β-sheets and random coils which is a core structural feature of BBIs (Figure 6; Voss et al., 1996; Qi et al., 2005; Banerjee et al., 2017). Also, the function of PnBBI in terms of its TI and CI activity was stable at a broad spectrum of pH and temperature, and corroborated well with its unaffected Far-UV CD spectra (Figures 5A,B, 6A,B). The structural features of PnBBI such as compactness, seven disulfide bonds and hydrogen-bonding network possibly aided stability to PnBBI under different adverse conditions (Tamura et al., 1994; Zavodszky et al., 2001; Zhou et al., 2005; Joshi et al., 2013). In contrast, the structural and functional stability of PnBBI was greatly affected by reducing agent DTT, particularly in its TI activity as compared to CI activity (Figures 5C, 6C). Also, the comparative resistance of its CI activity toward DTT reduction was found to be higher than BBIs from *Cajanus cajan* (Prasad et al., 2010c) and lower than rRsBBI from *R. sublobata* (Mohanraj et al., 2018). This could be due to the fact that disulfide bonds stabilizing the CI loop might have been buried and inaccessible to DTT as compared to trypsin reactive site loop structure of PnBBI (He et al., 2017). Also, the observed characteristics such as low molecular mass, the existence of several isoinhibitors in a self-association pattern, presence of TI and CI activity, typical secondary structural components and MALDI-MS/MS analysis identified the purified protein as BBI (Figures 1C, 3A,B, 6D, 7A).

### PnBBI Feeding Influenced the Growth of *H. armigera* by Modulating HaGPs Expression and Activity

*H. armigera* uses multiple defense strategies while feeding upon various host plant material. The digestive proteases and their flexibility in expression play a prime role in contributing a major support for its survival, adaptability and polyphagous nature (Padul et al., 2012; Macedo et al., 2015; Zhu-Salzman and Zeng, 2015; War et al., 2018; Jamal et al., 2019). At any given time, alkaline trypsin-like serine proteases are the major constituents of midgut, inspite of changes in digestive protease profile with developmental stage and type of diet it consumes. In this scenario, targeting these proteases with PnBBI which possessed significant inhibitory activity against HaTPs would be a potential approach to control the growth of larvae (Figure 7A; Harsulkar et al., 1999; Chougule et al., 2005; Srinivasan et al., 2006; Parde et al., 2010). The functional stability of PnBBI toward diverse pH conditions is well suited to adapt itself in the alkaline (pH 10-11) gut environment of *H. armigera* (Figure 5A). Hence, the present study was undertaken to evaluate the *ex vivo* and indigenous PnBBI effects on modulation in expression profiles of trypsin-like and chymotrypsin-like gut proteases, their activity, and associated changes in larval growth.

The strong insecticidal potential of PnBBI was primarily represented by low IC_50_ values (40 ng/ml) and high maximal inhibition of HaTPs by 86% (data not shown) as compared to the other reported PIs (Table 2; Babu and Subrahmanyam, 2010; Jamal et al., 2014; Pandey et al., 2014; Swathi et al., 2016; Banerjee et al., 2017; Mohanraj et al., 2019). Hence, the antifeedant effect of PnBBI against *H. armigera* at morphological as well as proteases expression level was further examined by biochemical and molecular approaches. In the present study, a significant reduction (40-50%) in larval body weight was observed with the intake of high dose PnBBI (0.005%). These observations are in accordance with the *in vitro* studies emphasizing the efficacy of PnBBI in inhibiting midgut proteases which in turn led to impaired protein digestion and hindered larval growth (Figures 7B–D). The modulation in expression of various digestive protease profiles with respect to diet intake have great significance for adaptation and survival of the insect (Chougule et al., 2005). The observed results are in agreement with the earlier reports (Harsulkar et al., 1999; Patankar et al., 2001; Telang et al., 2003; Chougule et al., 2005). Also, the HaTPI potential of PnBBI in limiting larval growth is further evident by loss in HaTP activity (63%) in larvae fed on a high dose of PnBBI (Figure 7D). The above observation was further confirmed by one and two-dimensional zymography. The comparative HaGP profiles on native-PAGE zymogram revealed the depletion of several trypsin-like proteases in larvae fed on PnBBI diet (Figures 8A–D). However, the proteases represented as group-II (6-10) have not shown any changes in their expression in response to PnBBI except a slight increase in the intensity of band 9 (Figure 8D). The inhibition in the activity of protease isoform 9 suggests that it belong to chymotrypsin-like protease and its increased expression might be compensatory response to minimize the effect of the PnBBI. Interestingly the disappeared proteases which belong to trypsin-type were inhibited by the PnBBI. However, remaining protease bands (6-10) were neither inhibited nor changed in their expression levels in response to PnBBI. The PnBBI mediated modulation in gut protease activity of *H. armigera* larvae was further supported by serine protease transcripts analysis using qRT-PCR. PnBBI influenced the expression of HaTP and HaCP transcripts in a dose- and exposure time-dependent manner. At any given time interval, the larvae exposed to low and moderate dose of PnBBI exhibited a similar pattern in expression of HaTP and HaCP transcripts when compared with those fed on a high dose of PnBBI (Figures 9A,B). Overall, the low and moderate dose of PnBBI ingestion regulated HaTP/HaCP transcripts by a signature pattern of down-up-down expression after feeding on day 3, 6 and 9, respectively. Conversely, a high dose of PnBBI fed larvae exhibited a pattern of up-down-up expression on day 3, 6 and 9, respectively, with few exceptions. Thus the expression pattern of trypsin- and chymotrypsin-like proteases in response to PnBBI ingestion was similar to the mode of trypsin expression in response to rCanPI-7 ingestion (Mahajan et al., 2013).

## Conclusion

In the present study, trypsin specific PI (PnBBI) was purified from the interspecific advanced peanut variety and confirmed as BBI by MALDI-MS-MS analysis. The purified PnBBI has low molecular mass with prominent TI activity and existed as several isoinhibitors with self-association tendency. The structural and functional integrity of PnBBI against a broad range of pH and temperature is advantageous to use PnBBI as a biopesticide in resilience with pest midgut alkaline microenvironment and temperature fluctuations in the field climate. The *in vitro* and *in vivo* studies demonstrated the TI potentiality of PnBBI in modulating the pest gut protease profiles which in turn would be helpful to understand the strategic application of PnBBI as a promising biocontrol agent in the management of *H. armigera*. Thus, this study provides a biochemical and molecular understanding on “HaTP mediated insecticidal potential of PnBBI from interspecific advanced peanut hybrid variety in combating agronomically important pest *H. armigera*.”

## Data Availability Statement

The protein identification data by (MALDI MS-MS) analysis can be found in ProteomeXchange consortium via PRIDE (Identifier PXD016933).

## Author Contributions

VL carried out purification, one and two-dimensional gel electrophoresis, biacore surface plasmon resonance, activity assays, CD and MALDI studies, *in vivo* antifeedant assays, qRT-PCR studies and manuscript writing. MS performed *in vitro* assays and contributed partially to several experiments shown above and manuscript writing. KP conceived and designed the experiments, supervised the experimental work, data analysis, manuscript editing, and critical review. NM performed generation of experimental material and maintenance, and critical review of the manuscript.

## Conflict of Interest

The authors declare that the research was conducted in the absence of any commercial or financial relationships that could be construed as a potential conflict of interest.

## Abbreviations

2-DE: Two-dimensional gel electrophoresis
BAPNA: N- α-benzoyl -DL-arginine-p-nitroanilide
CD: Circular dichroism
CI: Chymotrypsin inhibitor
DTT: Dithiothreitol
GLUPHEPA: N-glutaryl -L-phenylalanine-p-nitroanilide
Ha: H. armigera midgut chymotrypsin-like proteases
HaChy: H. armigera midgut chymotrypsin gene
HaCPI: H. armigera midgut chymotrypsin-like protease inhibitor
HaGPs: H. armigera midgut proteases
HaTPI: H. armigera midgut trypsin-like protease inhibitor
HaTPs: H. armigera midgut trypsin-like proteases
HaTry: H. armigera midgut trypsin gene
IDA: Iodoacetamide
IEF: Isoelectric focusing
KD: Equilibrium dissociation constant
MALDI-TOF: Matrix-assisted laser desorption ionization time-of-flight
PnBBI: Peanut Bowman-Birk inhibitors
PnCPI: peanut crude proteinase inhibitor extract
qRT-PCR: Quantitative real-time polymerase chain reaction
RU: Resonance unit
SPR: Surface plasmon resonance
TI: Trypsin inhibitor.
