# Supplementary figures and images for: Response of Midgut Trypsin- and Chymotrypsin-Like Proteases of Helicoverpa armigera Larvae Upon Feeding With Peanut BBI: Biochemical and Biophysical Characterization of PnBBI
Source: Front Plant Sci. 2020 Mar 24;11:266. doi: 10.3389/fpls.2020.00266 (PMC7105688; doi:10.3389/fpls.2020.00266)

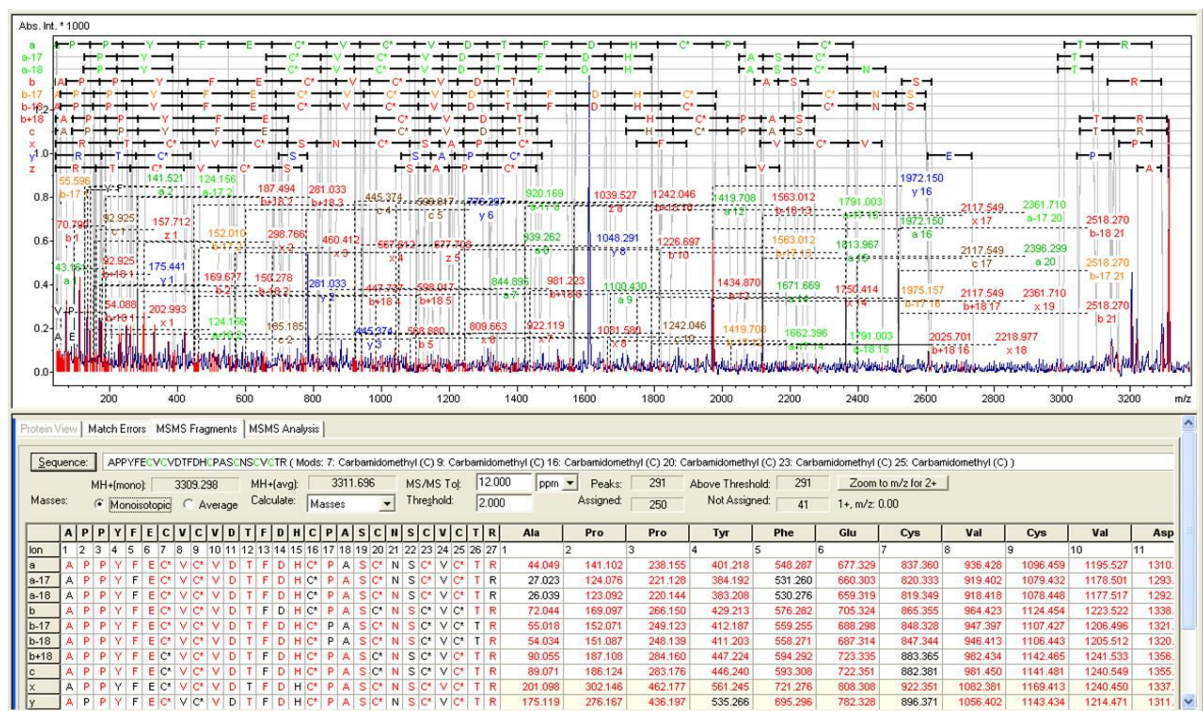

Zoomed spectrum of Supplementary Figure 1C

Supplement: Supplementary file 1 [file Image_1.pdf]
