# Supplementary material for: Response of Midgut Trypsin- and Chymotrypsin-Like Proteases of Helicoverpa armigera Larvae Upon Feeding With Peanut BBI: Biochemical and Biophysical Characterization of PnBBI
Source: Front Plant Sci. 2020 Mar 24;11:266. doi: 10.3389/fpls.2020.00266 (PMC7105688; doi:10.3389/fpls.2020.00266)
Supplement: Supplementary file 2 [file Data_Sheet_1.PDF]

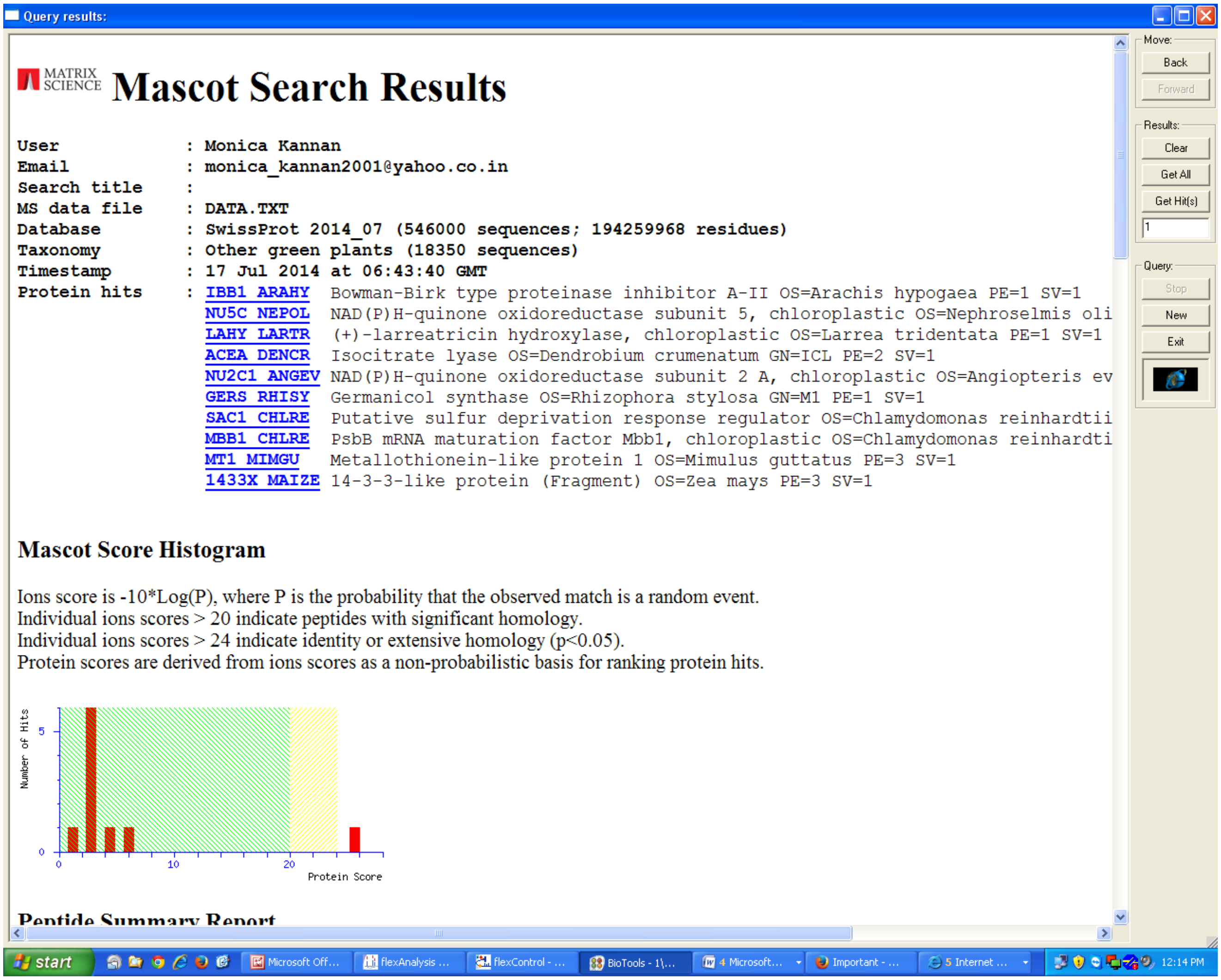
Query results:

Mascot Search Results

User : Monica Kannan
Email : monica_kannan2001@yahoo.co.in
Search title :
MS data file : DATA.TXT
Database : SwissProt 2014_07 (546000 sequences; 194259968 residues)
Taxonomy : Other green plants (18350 sequences)
Timestamp : 17 Jul 2014 at 06:43:40 GMT
Protein hits : IBB1_ARAHY Bowman-Birk type proteinase inhibitor A-II OS=Arachis hypogaea PE=1 SV=1
NU5C_NEPOL NAD(P)H-quinone oxidoreductase subunit 5, chloroplastic OS=Nephroselmis oli
LAHY_LARTR (+)-larreatricin hydroxylase, chloroplastic OS=Larrea tridentata PE=1 SV=1
ACEA_DENCR Isocitrate lyase OS=Dendrobium crumenatum GN=ICL PE=2 SV=1
NU2C1_ANGEV NAD(P)H-quinone oxidoreductase subunit 2 A, chloroplastic OS=Angiopteris ev
GERS_RHISY Germanicol synthase OS=Rhizophora stylosa GN=M1 PE=1 SV=1
SAC1_CHLRE Putative sulfur deprivation response regulator OS=Chlamydomonas reinhardtii
MBB1_CHLRE PsbB mRNA maturation factor Mbb1, chloroplastic OS=Chlamydomonas reinhardti
MT1_MIMGU Metallothionein-like protein 1 OS=Mimulus guttatus PE=3 SV=1
1433X_MAIZE 14-3-3-like protein (Fragment) OS=Zea mays PE=3 SV=1

Mascot Score Histogram

Ions score is -10*Log(P), where P is the probability that the observed match is a random event.
Individual ions scores > 20 indicate peptides with significant homology.
Individual ions scores > 24 indicate identity or extensive homology (p<0.05).
Protein scores are derived from ions scores as a non-probabilistic basis for ranking protein hits.

Peptide Summary Report

. [IBB1_ARAHY](#) **Mass:** 8426 **Score:** 26 **Matches:** 1(1) **Sequences:** 1(1)

Bowman-Birk type proteinase inhibitor A-II OS=Arachis hypogaea PE=1 SV=1

☐ Check to include this hit in error tolerant search

Query	Observed	Mr(expt)	Mr(calc)	ppm	Miss	Score	Expect	Rank	Unique	Peptide
1	3313.8630	3312.8557	3308.2913	1380	0	26	0.042	1	U	R.APPYFECVCVDTFDHCPASCNSCVCTR.S

Protein View: IBB1_ARAHY

Bowman-Birk type proteinase inhibitor A-II OS=Arachis hypogaea PE=1 SV=1

Database: SwissProt

Score: 26

Nominal mass (M_r): 8426

Calculated pI: 5.08

Taxonomy: Arachis hypogaea

Sequence similarity is available as an NCBI BLAST search of IBB1_ARAHY against nr.

Search parameters

MS data file: DATA.TXT

Enzyme: Trypsin: cuts C-term side of KR unless next residue is P.

Fixed modifications: Carbamidomethyl (C)

Variable modifications: Oxidation (M)

Protein sequence coverage: 38%

Matched peptides shown in ***bold red***.

```
 1  EASSSSDDNV  CCNGCLCDRR  APPYFECVCV  DTFDHCPASC  NSCVCTRSNP
51  PQCRCTDKTQ  GRCPVTECRS
```

Unformatted sequence string: 70 residues (for pasting into other applications).

```
AC   P01066;
DT   21-JUL-1986, integrated into UniProtKB/Swiss-Prot.
DT   21-JUL-1986, sequence version 1.
DT   16-APR-2014, entry version 68.
DE   RecName: Full=Bowman-Birk type proteinase inhibitor A-II;
DE   Contains:
DE     RecName: Full=Bowman-Birk type proteinase inhibitor A-I;
DE   Contains:
DE     RecName: Full=Bowman-Birk type proteinase inhibitor B-I;
DE   Contains:
DE     RecName: Full=Bowman-Birk type proteinase inhibitor B-III;
OS   Arachis hypogaea (Peanut).
OC   Eukaryota; Viridiplantae; Streptophyta; Embryophyta; Tracheophyta;
OC   Spermatophyta; Magnoliophyta; eudicotyledons; Gunneridae;
OC   Pentapetalae; rosids; fabids; Fabales; Fabaceae; Papilionoideae;
OC   Dalbergieae; Arachis.
OX   NCBI_TaxID=3818;
RN   [1]
RP   PROTEIN SEQUENCE OF 1-19.
RX   PubMed=6630176;
RA   Norioka S., Ikenaka T.;
RT   "Amino acid sequences of trypsin-chymotrypsin inhibitors (A-I, A-II,
RT   B-I, and B-II) from peanut (Arachis hypogaea): a discussion on the
RT   molecular evolution of legume Bowman-Birk type inhibitors.";
RL   J. Biochem. 94:589-599(1983).
RN   [2]
RP   PROTEIN SEQUENCE OF 10-70.
RX   PubMed=6841347;
RA   Norioka S., Ikenaka T.;
RT   "Amino acid sequence of a trypsin-chymotrypsin inhibitor, B-III, of
RT   peanut (Arachis hypogaea).";
RL   J. Biochem. 93:479-485(1983).
RN   [3]
RP   X-RAY CRYSTALLOGRAPHY (3.3 ANGSTROMS).
RX   PubMed=3571206;
RA   Suzuki A., Tsunogae Y., Tanaka I., Yamane T., Ashida T., Norioka S.,
RA   Hara S., Ikenaka T.;
RT   "The structure of Bowman-Birk type protease inhibitor A-II from peanut
RT   (Arachis hypogaea) at 3.3-A resolution.";
RL   J. Biochem. 101:267-274(1987).
RN   [4]
RP   X-RAY CRYSTALLOGRAPHY (2.3 ANGSTROMS).
RX   PubMed=8254669; DOI=10.1006/jmbi.1993.1622;
RA   Suzuki A., Yamane T., Ashida T., Norioka S., Hara S., Ikenaka T.;
RT   "Crystallographic refinement of Bowman-Birk type protease inhibitor A-
RT   II from peanut (Arachis hypogaea) at 2.3-A resolution.";
RL   J. Mol. Biol. 234:722-734(1993).
CC   -!- FUNCTION: These proteins inhibit trypsin and chymotrypsin, having
CC       2 sites of interaction with trypsin. The site of interaction with
CC       chymotrypsin has not been determined but is not independent of the
CC       trypsin-reactive sites.
CC   -!- MISCELLANEOUS: Four inhibitors were found that are identical
CC       except at their amino ends and that probably arise by proteolytic
CC       degradation of a single gene product.
CC   -!- SIMILARITY: Belongs to the Bowman-Birk serine protease inhibitor
CC       family.
CC   -----------------------------------------------------------------------
```

```
CC   Copyrighted by the UniProt Consortium, see http://www.uniprot.org/terms
CC   Distributed under the Creative Commons Attribution-NoDerivs License
CC   ---------------------------------------------------------------------------
DR   PIR; A91975; TINPA2.
DR   ProteinModelPortal; P01066; -.
DR   MEROPS; I12.006; -.
DR   GO; GO:0005576; C:extracellular region; IEA:InterPro.
DR   GO; GO:0004867; F:serine-type endopeptidase inhibitor activity;
IEA:UniProtKB-KW.
DR   Gene3D; 2.10.69.10; -; 1.
DR   InterPro; IPR000877; Prot_inh_BBI.
DR   Pfam; PF00228; Bowman-Birk_leg; 1.
DR   SMART; SM00269; BowB; 1.
DR   SUPFAM; SSF57247; SSF57247; 1.
DR   PROSITE; PS00281; BOWMAN_BIRK; 1.
PE   1: Evidence at protein level;
KW   Direct protein sequencing; Disulfide bond; Protease inhibitor;
KW   Serine protease inhibitor.
FT   CHAIN         1     70       Bowman-Birk type proteinase inhibitor A-
FT                                II.
FT                                /FTId=PRO_0000003260.
FT   CHAIN         4     70       Bowman-Birk type proteinase inhibitor A-
FT                                I.
FT                                /FTId=PRO_0000003261.
FT   CHAIN         8     70       Bowman-Birk type proteinase inhibitor B-
FT                                I.
FT                                /FTId=PRO_0000003262.
FT   CHAIN        10     70       Bowman-Birk type proteinase inhibitor B-
FT                                III.
FT                                /FTId=PRO_0000003263.
FT   SITE         19     20       Reactive bond for trypsin.
FT   SITE         47     48       Reactive bond for trypsin.
FT   DISULFID     11     68
FT   DISULFID     12     29
FT   DISULFID     15     63
FT   DISULFID     17     27
FT   DISULFID     36     43
FT   DISULFID     40     55
FT   DISULFID     45     53
SQ   SEQUENCE   70 AA;  7633 MW;  56CE31B690015CD5 CRC64;
     EASSSSDDNV CCNGCLCDRR APPYFECVCV DTFDHCPASC NSCVCTRSNP PQCRCTDKTQ
     GRCPVTECRS
```

http://www.matrixscience.com/cgi/protein_view.pl?file=..%2Fdata%2F20140717%2FFTgtSiYaO.dat&hit=IBB1_ARAHY&db_idx=1&px=1&ave_thresh=24&_ignoreionsscorebelow=0&report=10&_sigthreshold=0.05&_msresflags=1025&_msresflags2=2&percolate=-1&percolate_rt=0&_minpeplen=7&sessionID=guest_guestsession

Search Parameters

```
Type of search         : MS/MS Ion Search
Enzyme                 : Trypsin
Fixed modifications    : Carbamidomethyl (C)
Variable modifications : Oxidation (M)
Mass values            : Monoisotopic
Protein Mass           : Unrestricted
Peptide Mass Tolerance : ± 1379.9 ppm
Fragment Mass Tolerance: ± 1.2 Da
Max Missed Cleavages   : 0
Instrument type        : MALDI-TOF-TOF
Number of queries      : 1
```
