# Supplementary material for: Response of Midgut Trypsin- and Chymotrypsin-Like Proteases of Helicoverpa armigera Larvae Upon Feeding With Peanut BBI: Biochemical and Biophysical Characterization of PnBBI
Source: Front Plant Sci. 2020 Mar 24;11:266. doi: 10.3389/fpls.2020.00266 (PMC7105688; doi:10.3389/fpls.2020.00266)
Supplement: Supplementary file 3 [file Data_Sheet_2.PDF]

Mascot Search Results

```
User          : Monica Kannan
Email         : monica_kannan2001@yahoo.co.in
Search title  :
MS data file  : DATA.TXT
Database      : SwissProt 57.15 (515203 sequences; 181334896 residues)
Taxonomy      : Other green plants (16973 sequences)
Timestamp     : 31 Dec 2019 at 06:44:11 GMT
Protein hits  : IBB1_ARAHY  Bowman-Birk type proteinase inhibitor A-II OS=Arachis hypogaea PE=1 SV=1
                RPOB_PINKO  DNA-directed RNA polymerase subunit beta OS=Pinus koraiensis GN=rpoB PE=3 SV=1
                NU5C_NEPOL  NAD(P)H-quinone oxidoreductase subunit 5, chloroplastic OS=Nephroselmis olivacea GN=ndhF PE=3 SV=1
                RPOB_HELSJ  DNA-directed RNA polymerase subunit beta OS=Helicosporidium sp. subsp. Simulium jonesii GN=rpoB PE=3 SV=1
                DRTS_SOYBN  Bifunctional dihydrofolate reductase-thymidylate synthase OS=Glycine max PE=1 SV=1
                NU2C1_ANGEV NAD(P)H-quinone oxidoreductase subunit 2 A, chloroplastic OS=Angiopteris evecta GN=ndhB1 PE=3 SV=1
                MBB1_CHLRE  PsbB mRNA maturation factor Mbb1, chloroplastic OS=Chlamydomonas reinhardtii GN=MBB1 PE=2 SV=1
                LEC1_DOLBI  Seed lectin subunit I OS=Dolichos biflorus PE=1 SV=2
                DYHG_CHLRE  Dynein gamma chain, flagellar outer arm OS=Chlamydomonas reinhardtii GN=ODA2 PE=1 SV=1
                1433X_MAIZE 14-3-3-like protein (Fragment) OS=Zea mays PE=3 SV=1
```

Mascot Score Histogram

Ions score is -10*Log(P), where P is the probability that the observed match is a random event.
Individual ions scores > 18 indicate peptides with significant homology.
Individual ions scores > 24 indicate identity or extensive homology (p<0.05).
Protein scores are derived from ions scores as a non-probabilistic basis for ranking protein hits.

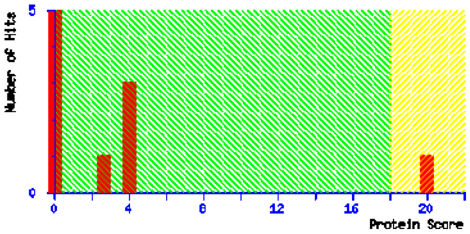

Peptide Summary Report

Format As: Peptide Summary | Help

Significance threshold p< 0.05 | Max. number of hits 10

Standard scoring ◉ MudPIT scoring ○ | Ions score or expect cut-off 0 | Show sub-sets 0

Show pop-ups ◉ Suppress pop-ups ○ | Sort unassigned Decreasing Score | Require bold red ☐

Overview Table

Click on column header to jump to entry in results list.
Move mouse over any indicator to highlight identical peptides.
Click on an indicator to see details of individual match.
Use check boxes to select sub-set of queries for new search.

Mouse over: -Query- | -Accession- | -Sequence-

Hit:	1	2	3	4	5	6	7	8	9	10
☑ 3313.8630 (1+)	●	●	●	●	●	●	●	●	●	●

Select All | Select None | Search Selected | ☐ **Error tolerant** | Archive Report

1. IBB1_ARAHY **Mass:** 8426 **Score:** 20 **Matches:** 1(0) **Sequences:** 1(0)
Bowman-Birk type proteinase inhibitor A-II OS=Arachis hypogaea PE=1 SV=1
☐ Check to include this hit in error tolerant search or archive report

Query	Observed	Mr(expt)	Mr(calc)	ppm	Miss	Score	Expect	Rank	Unique	Peptide
1	3313.8630	3312.8557	3308.2913	1380	0	20	0.13	1	U	R.APPYFECVCVDTFDHCPASCNSCVCTR.S

---

2. RPOB_PINKO **Score:** 5 **Matches:** 1(0) **Sequences:** 1(0)
DNA-directed RNA polymerase subunit beta OS=Pinus koraiensis GN=rpoB PE=3 SV=1
☐ Check to include this hit in error tolerant search or archive report

Query	Observed	Mr(expt)	Mr(calc)	ppm	Miss	Score	Expect	Rank	Unique	Peptide
1	3313.8630	3312.8557	3316.5902	-1125.99	0	5	4.4	2	U	K.NTFQDFFGSHPLSQFLDQTNPLTEIAHGR.K

Proteins matching the same set of peptides:
RPOB_PINTH **Score:** 5 **Matches:** 1(0) **Sequences:** 1(0)

---

3. NU5C_NEPOL **Mass:** 71741 **Score:** 4 **Matches:** 1(0) **Sequences:** 1(0)
NAD(P)H-quinone oxidoreductase subunit 5, chloroplastic OS=Nephroselmis olivacea GN=ndhF PE=3 SV=1
☐ Check to include this hit in error tolerant search or archive report

Query	Observed	Mr(expt)	Mr(calc)	ppm	Miss	Score	Expect	Rank	Unique	Peptide
1	3313.8630	3312.8557	3309.8536	907	0	4	4.7	3	U	R.LHGALAIGAMALSFVVSLGVLWNQLHGIAPVR.W

4. RPOB_HELSJ **Score:** 3 **Matches:** 1(0) **Sequences:** 1(0)
DNA-directed RNA polymerase subunit beta OS=Helicosporidium sp. subsp. Simulium jonesii GN=rpoB PE=3 SV=1
☐ Check to include this hit in error tolerant search or archive report

Query	Observed	Mr(expt)	Mr(calc)	ppm	Miss	Score	Expect	Rank	Unique	Peptide
1	3313.8630	3312.8557	3316.6550	-1145.52	0	3	5.8	4	U	K.ISFQSLSPYHMISLATSLIPFLEHNDANR.A + Oxidation (M)

5. DRTS_SOYBN **Score:** 2 **Matches:** 1(0) **Sequences:** 1(0)
Bifunctional dihydrofolate reductase-thymidylate synthase OS=Glycine max PE=1 SV=1
☐ Check to include this hit in error tolerant search or archive report

Query	Observed	Mr(expt)	Mr(calc)	ppm	Miss	Score	Expect	Rank	Unique	Peptide
1	3313.8630	3312.8557	3316.4749	-1091.26	0	2	7.4	6	U	K.LMALPPCHMFAQFYVAHGELSCQMYQR.S + 2 Oxidation (M)

6. NU2C1_ANGEV **Score:** 1 **Matches:** 1(0) **Sequences:** 1(0)
NAD(P)H-quinone oxidoreductase subunit 2 A, chloroplastic OS=Angiopteris evecta GN=ndhB1 PE=3 SV=1
☐ Check to include this hit in error tolerant search or archive report

Query	Observed	Mr(expt)	Mr(calc)	ppm	Miss	Score	Expect	Rank	Unique	Peptide
1	3313.8630	3312.8557	3309.7216	947	0	1	11	8	U	R.IVNGLLTTQMYNSTGMFISMIFLLVGVGFK.L + Oxidation (M)

Proteins matching the same set of peptides:
NU2C2_ANGEV **Score:** 1 **Matches:** 1(0) **Sequences:** 1(0)

7. MBB1_CHLRE **Score:** 1 **Matches:** 1(0) **Sequences:** 1(0)
PsbB mRNA maturation factor Mbb1, chloroplastic OS=Chlamydomonas reinhardtii GN=MBB1 PE=2 SV=1
☐ Check to include this hit in error tolerant search or archive report

Query	Observed	Mr(expt)	Mr(calc)	ppm	Miss	Score	Expect	Rank	Unique	Peptide
1	3313.8630	3312.8557	3312.6110	73.9	0	1	11	9	U	R.LVESNDLSALPDFLSSDDDVEASLRPPGAAGR.R

8. LEC1_DOLBI **Mass:** 29388 **Score:** 0 **Matches:** 1(0) **Sequences:** 1(0)
Seed lectin subunit I OS=Dolichos biflorus PE=1 SV=2
☐ Check to include this hit in error tolerant search or archive report

Query	Observed	Mr(expt)	Mr(calc)	ppm	Miss	Score	Expect	Rank	Unique	Peptide
1	3313.8630	3312.8557	3315.7788	-881.58	0	0	13	10	U	-.MASSTVSVVLSLFLLLLTQANSANIQSFSFK.N

9. DYHG_CHLRE **Score:** 0 **Matches:** 1(0) **Sequences:** 1(0)
Dynein gamma chain, flagellar outer arm OS=Chlamydomonas reinhardtii GN=ODA2 PE=1 SV=1
☐ Check to include this hit in error tolerant search or archive report

Query	Observed	Mr(expt)	Mr(calc)	ppm	Miss	Score	Expect	Rank	Unique	Peptide
1	3313.8630	3312.8557	3309.5257	1006	0	2	7.5	7	U	K.QMCIFVDDISMPYINEWGHQVTNEIVR.Q + Oxidation (M)

10. 1433X_MAIZE **Mass:** 6812 **Score:** 0 **Matches:** 1(0) **Sequences:** 1(0)
14-3-3-like protein (Fragment) OS=Zea mays PE=3 SV=1
☐ Check to include this hit in error tolerant search or archive report

Query	Observed	Mr(expt)	Mr(calc)	ppm	Miss	Score	Expect	Rank	Unique	Peptide
1	3313.8630	3312.8557	3315.5340	-807.81	0	3	6.5	5	U	K.DSTLIMQLLYDNLTLWTSDTNEDGGDEIK.- + Oxidation (M)

Search Parameters

Type of search : **MS/MS Ion Search**
Enzyme : **Trypsin**
Fixed modifications : **Carbamidomethyl (C)**
Variable modifications : **Oxidation (M)**
Mass values : **Monoisotopic**
Protein Mass : **Unrestricted**
Peptide Mass Tolerance : **± 1379.9 ppm**
Fragment Mass Tolerance: **± 1.2 Da**
Max Missed Cleavages : **0**
Instrument type : **MALDI-TOF-TOF**
Number of queries : **1**

Mascot: http://www.matrixscience.com/

Mascot Search Results

Protein View

Match to: **IBB1_ARAHY** Score: **20**
Bowman-Birk type proteinase inhibitor A-II OS=Arachis hypogaea PE=1 SV=1
Found in search of DATA.TXT

Nominal mass (M_r): **8426**; Calculated pI value: **5.08**
NCBI BLAST search of IBB1_ARAHY against nr
Unformatted sequence string for pasting into other applications

Taxonomy: Arachis hypogaea

Fixed modifications: Carbamidomethyl (C)
Variable modifications: Oxidation (M)
Cleavage by Trypsin: cuts C-term side of KR unless next residue is P
Sequence Coverage: **38%**

Matched peptides shown in **Bold Red**

```
 1 EASSSSDDNV CCNGCLCDRR APPYFECVCV DTFDHCPASC NSCVCTRSNP
51 PQCRCTDKTQ GRCPVTECRS
```

Show predicted peptides also

Sort Peptides By ◉ Residue Number ○ Increasing Mass ○ Decreasing Mass

Start - End	Observed	Mr(expt)	Mr(calc)	ppm	Miss	Sequence	
21 - 47	3313.8630	3312.8557	3308.2913	1380	0	R.APPYFECVCVDTFDHCPASCNSCVCTR.S	(Ions score 20)

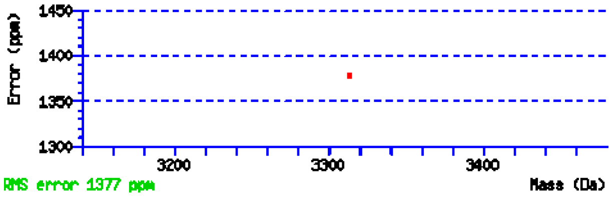


```
ID   IBB1_ARAHY              Reviewed;         70 AA.
AC   P01066;
DT   21-JUL-1986, integrated into UniProtKB/Swiss-Prot.
DT   21-JUL-1986, sequence version 1.
DT   02-MAR-2010, entry version 58.
DE   RecName: Full=Bowman-Birk type proteinase inhibitor A-II;
DE   Contains:
DE     RecName: Full=Bowman-Birk type proteinase inhibitor A-I;
DE   Contains:
DE     RecName: Full=Bowman-Birk type proteinase inhibitor B-I;
DE   Contains:
DE     RecName: Full=Bowman-Birk type proteinase inhibitor B-III;
OS   Arachis hypogaea (Peanut).
OC   Eukaryota; Viridiplantae; Streptophyta; Embryophyta; Tracheophyta;
OC   Spermatophyta; Magnoliophyta; eudicotyledons; core eudicotyledons;
OC   rosids; fabids; Fabales; Fabaceae; Papilionoideae; Dalbergieae;
OC   Arachis.
OX   NCBI_TaxID=3818;
RN   [1]
RP   PROTEIN SEQUENCE OF 1-19.
RX   MEDLINE=84032360; PubMed=6630176;
RA   Norioka S., Ikenaka T.;
RT   "Amino acid sequences of trypsin-chymotrypsin inhibitors (A-I, A-II,
RT   B-I, and B-II) from peanut (Arachis hypogaea): a discussion on the
RT   molecular evolution of legume Bowman-Birk type inhibitors.";
RL   J. Biochem. 94:589-599(1983).
RN   [2]
RP   PROTEIN SEQUENCE OF 10-70.
RX   MEDLINE=83186120; PubMed=6841347;
RA   Norioka S., Ikenaka T.;
RT   "Amino acid sequence of a trypsin-chymotrypsin inhibitor, B-III, of
RT   peanut (Arachis hypogaea).";
RL   J. Biochem. 93:479-485(1983).
RN   [3]
RP   X-RAY CRYSTALLOGRAPHY (3.3 ANGSTROMS).
RX   MEDLINE=87194702; PubMed=3571206;
RA   Suzuki A., Tsunogae Y., Tanaka I., Yamane T., Ashida T., Norioka S.,
RA   Hara S., Ikenaka T.;
RT   "The structure of Bowman-Birk type protease inhibitor A-II from peanut
RT   (Arachis hypogaea) at 3.3-A resolution.";
RL   J. Biochem. 101:267-274(1987).
RN   [4]
RP   X-RAY CRYSTALLOGRAPHY (2.3 ANGSTROMS).
RX   MEDLINE=94076346; PubMed=8254669; DOI=10.1006/jmbi.1993.1622;
```

```
RA   Suzuki A., Yamane T., Ashida T., Norioka S., Hara S., Ikenaka T.;
RT   "Crystallographic refinement of Bowman-Birk type protease inhibitor A-
RT   II from peanut (Arachis hypogaea) at 2.3-A resolution.";
RL   J. Mol. Biol. 234:722-734(1993).
CC   -!- FUNCTION: These proteins inhibit trypsin and chymotrypsin, having
CC       2 sites of interaction with trypsin. The site of interaction with
CC       chymotrypsin has not been determined but is not independent of the
CC       trypsin-reactive sites.
CC   -!- MISCELLANEOUS: Four inhibitors were found that are identical
CC       except at their amino ends and that probably arise by proteolytic
CC       degradation of a single gene product.
CC   -!- SIMILARITY: Belongs to the Bowman-Birk serine protease inhibitor
CC       family.
CC   -----------------------------------------------------------------------
CC   Copyrighted by the UniProt Consortium, see http://www.uniprot.org/terms
CC   Distributed under the Creative Commons Attribution-NoDerivs License
CC   -----------------------------------------------------------------------
DR   PIR; A91975; TINPA2.
DR   SMR; P01066; 9-60.
DR   MEROPS; I12.006; -.
DR   MEROPS; I12.017; -.
DR   GO; GO:0005576; C:extracellular region; IEA:InterPro.
DR   GO; GO:0004867; F:serine-type endopeptidase inhibitor activity; IEA:UniProtKB-KW.
DR   InterPro; IPR000877; Prot_inh_BBI.
DR   Gene3D; G3DSA:2.10.69.10; Prot_inh_BBI; 1.
DR   Pfam; PF00228; Bowman-Birk_leg; 1.
DR   SMART; SM00269; BowB; 1.
DR   SUPFAM; SSF57247; Prot_inh_BBI; 1.
DR   PROSITE; PS00281; BOWMAN_BIRK; 1.
PE   1: Evidence at protein level;
KW   Direct protein sequencing; Disulfide bond; Protease inhibitor;
KW   Serine protease inhibitor.
FT   CHAIN         1     70       Bowman-Birk type proteinase inhibitor A-
FT                                II.
FT                                /FTId=PRO_0000003260.
FT   CHAIN         4     70       Bowman-Birk type proteinase inhibitor A-
FT                                I.
FT                                /FTId=PRO_0000003261.
FT   CHAIN         8     70       Bowman-Birk type proteinase inhibitor B-
FT                                I.
FT                                /FTId=PRO_0000003262.
FT   CHAIN        10     70       Bowman-Birk type proteinase inhibitor B-
FT                                III.
FT                                /FTId=PRO_0000003263.
FT   SITE         19     20       Reactive bond for trypsin.
FT   SITE         47     48       Reactive bond for trypsin.
FT   DISULFID     11     68
FT   DISULFID     12     29
FT   DISULFID     15     63
FT   DISULFID     17     27
FT   DISULFID     36     43
FT   DISULFID     40     55
FT   DISULFID     45     53
SQ   SEQUENCE   70 AA;  7633 MW;  56CE31B690015CD5 CRC64;
     EASSSSDDNV CCNGCLCDRR APPYFECVCV DTFDHCPASC NSCVCTRSNP PQCRCTDKTQ
     GRCPVTECRS
```

Mascot: http://www.matrixscience.com/
