# Supplementary material for: Response of Midgut Trypsin- and Chymotrypsin-Like Proteases of Helicoverpa armigera Larvae Upon Feeding With Peanut BBI: Biochemical and Biophysical Characterization of PnBBI
Source: Front Plant Sci. 2020 Mar 24;11:266. doi: 10.3389/fpls.2020.00266 (PMC7105688; doi:10.3389/fpls.2020.00266)
Supplement: Supplementary file 4 [file Data_Sheet_3.PDF]

(duplicate) (triplicate)

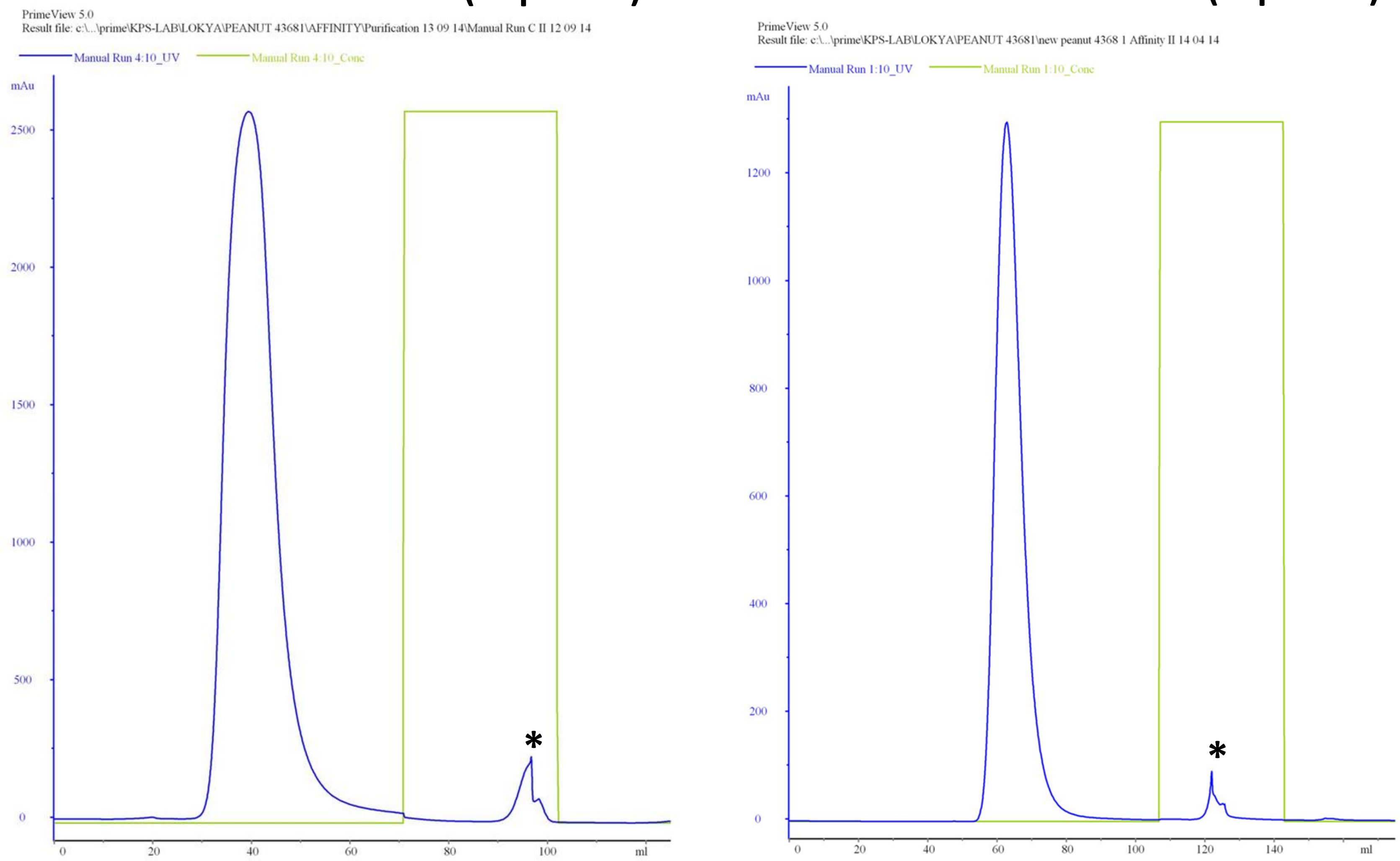

Figure 1A: Purification profile of PnBBI. Elution profile of trypsin-Sepharose 4B column loaded with 20-60% $(NH_4)_2SO_4$ active fraction. Asterisks indicate active peak with inhibitory activity against trypsin.

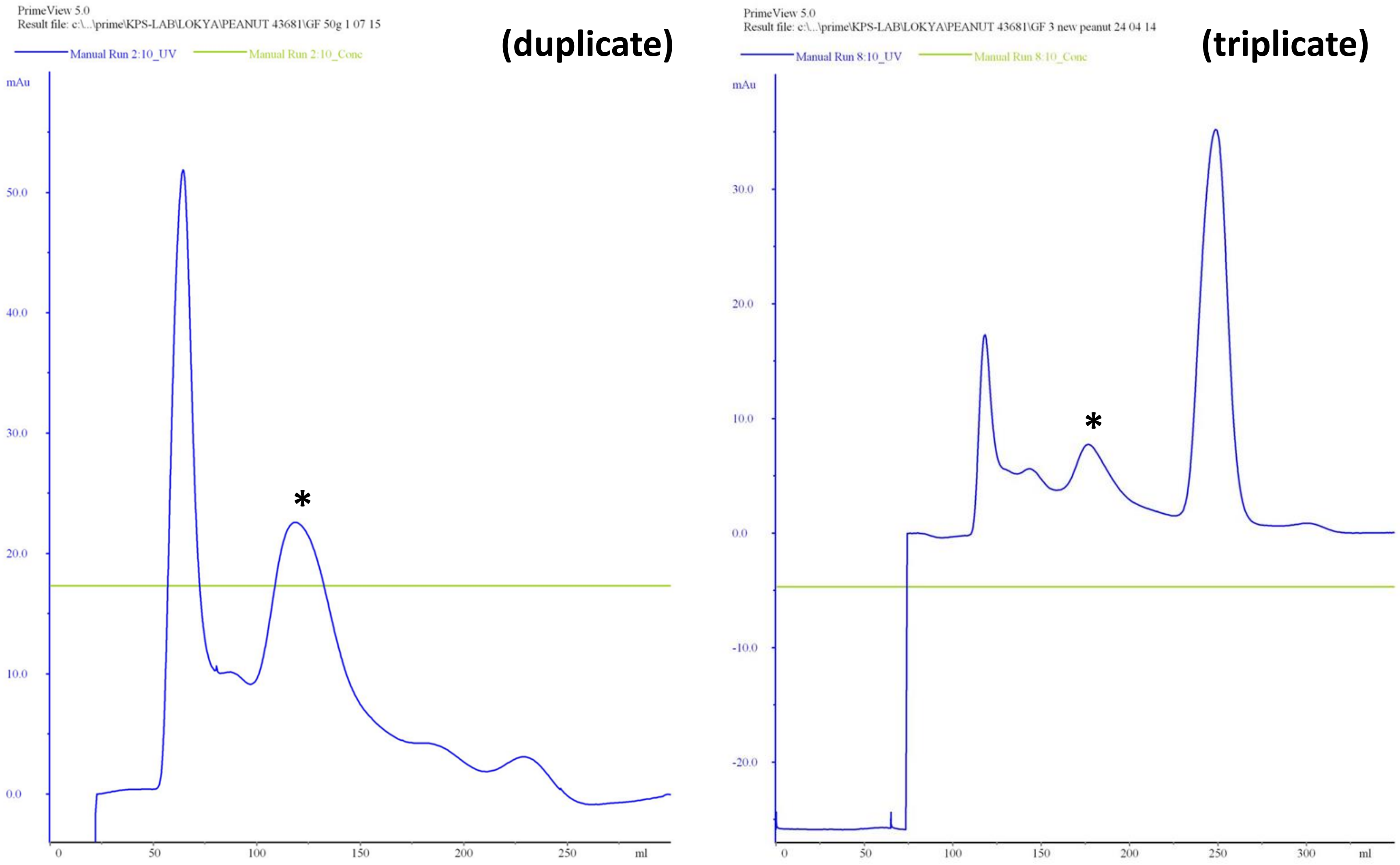

Figure 1B: Purification profile of PnBBI. Elution profile of Sephadex G-50 fine column loaded with active peak fraction pool of trypsin affinity column. Asterisks indicate active peak with inhibitory activity against trypsin.

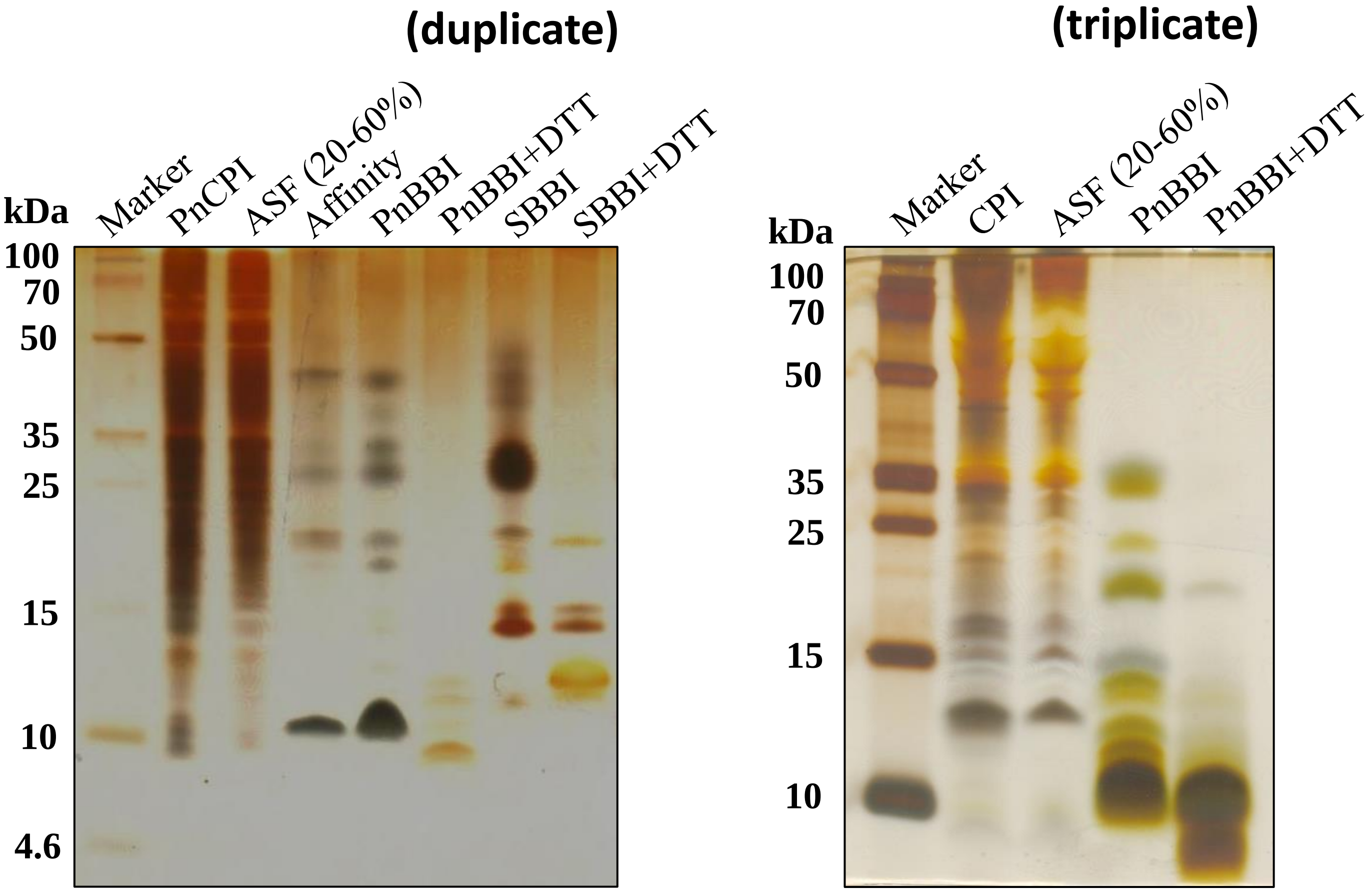

Figure 1C: Tricine SDS-PAGE (15%) showing purification profile and self-association pattern of PnBBI (**PnCPI**-crude proteinase inhibitor extract; **ASF**-ammonium sulfate fraction.

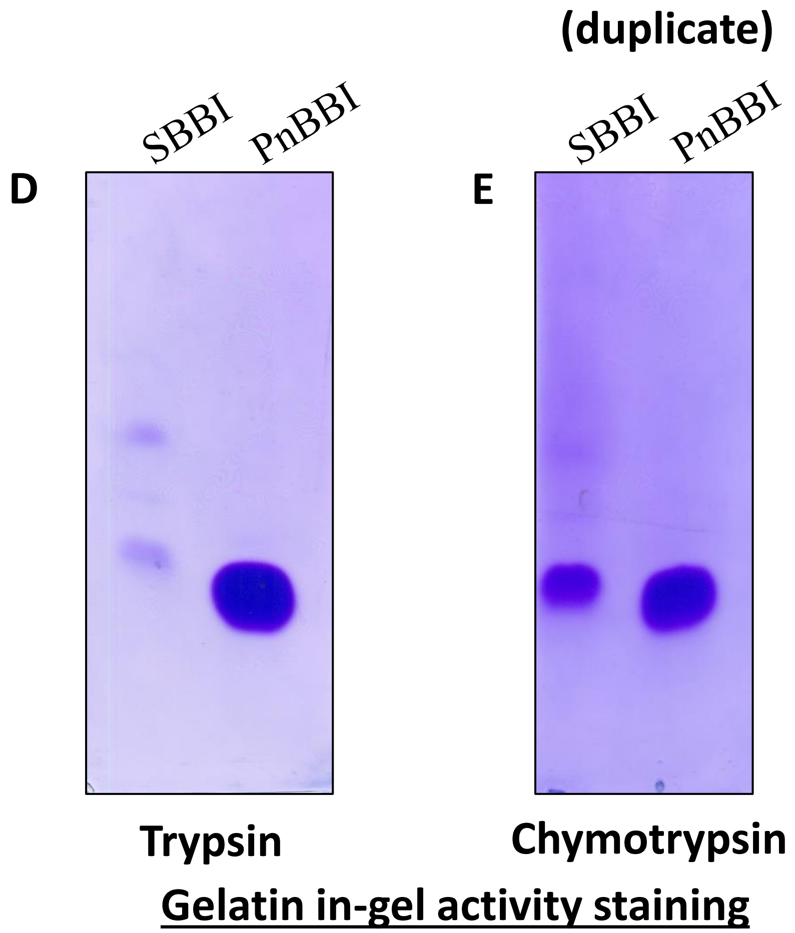

Figure 1D & E: PnBBI active against bovine pancreatic trypsin and chymotrypsin.

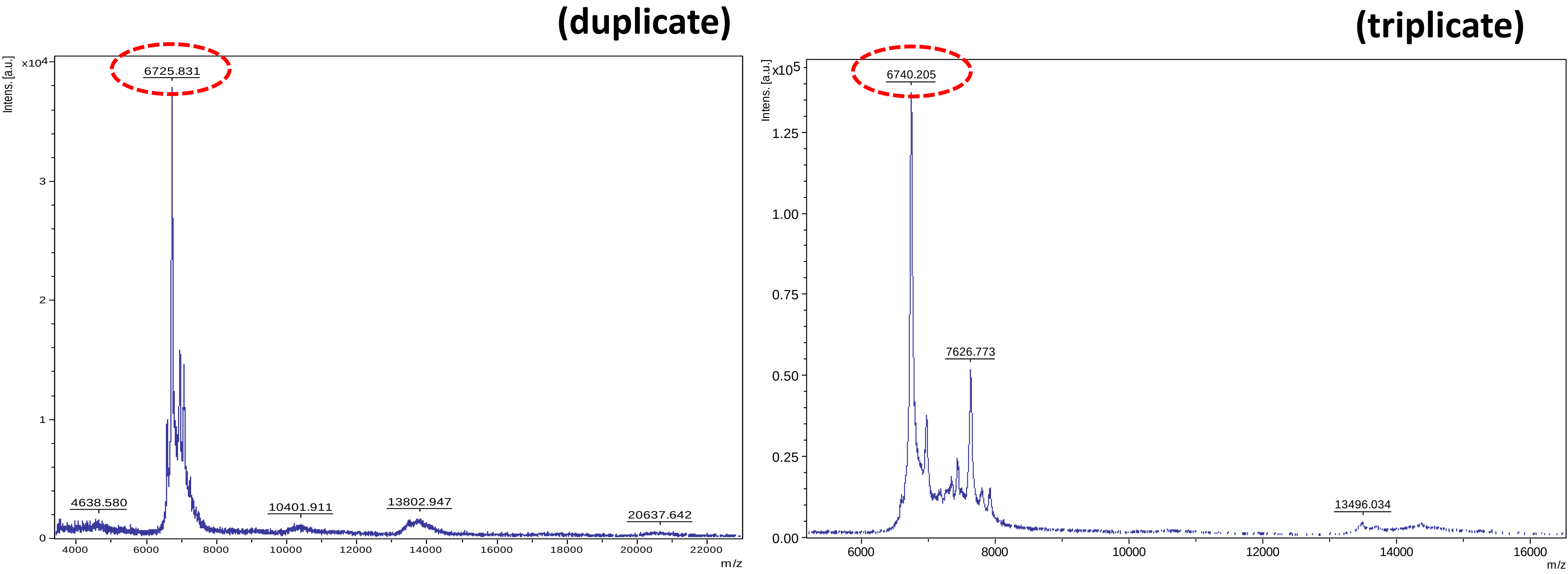

Figure 2A: MALDI-TOF spectrum of intact PnBBI.

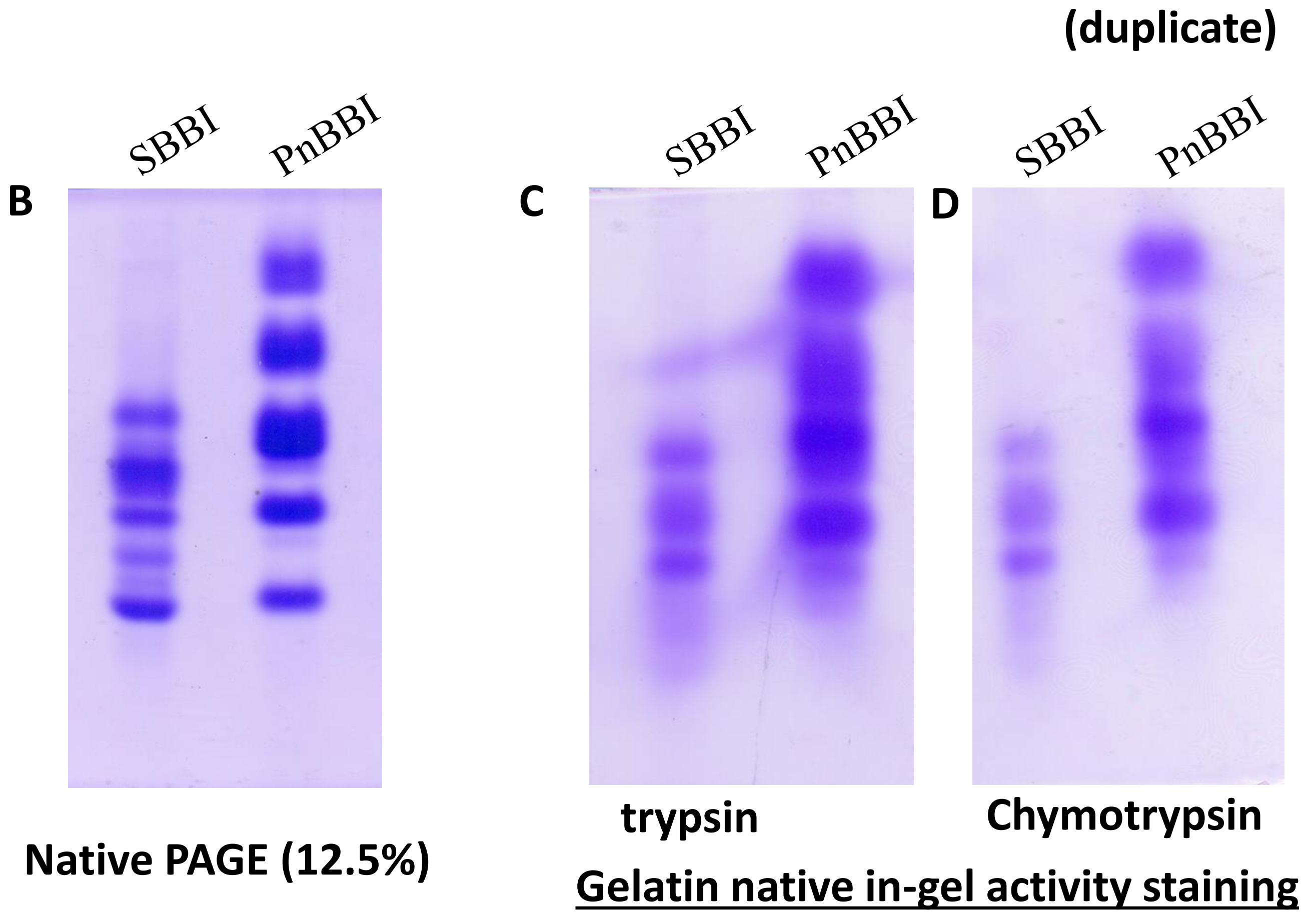

Figure 2B-D: (B) Visualization of PnBBI isoinhibitors resolved on native gel electrophoresis (12.5%). In-gel activity staining against bovine **(C)** trypsin and **(D)** chymotrypsin resolved on gelatin Native-PAGE.

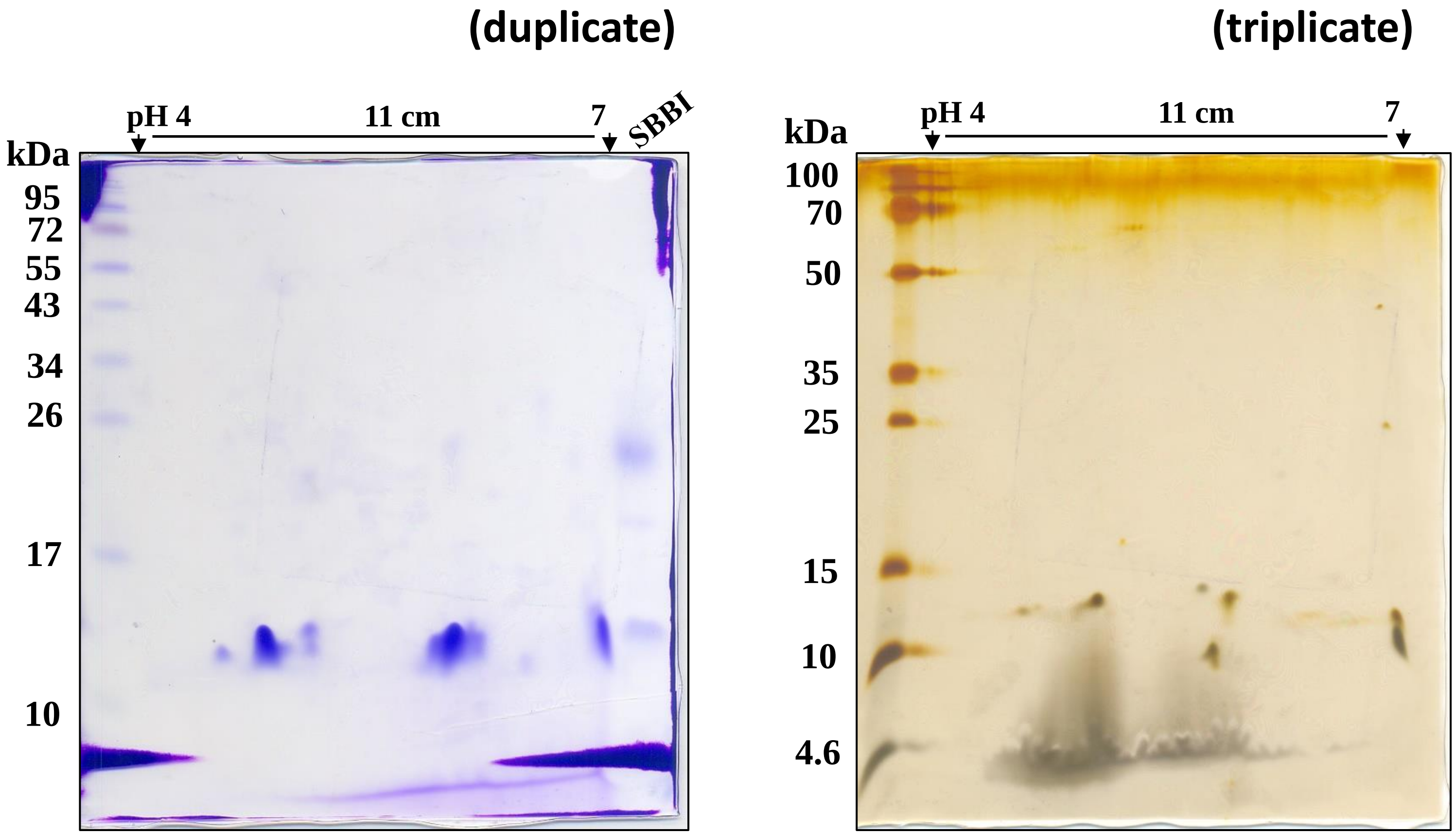

Figure 2E. 2-D gel electrophoresis of PnBBI under non-reducing conditions

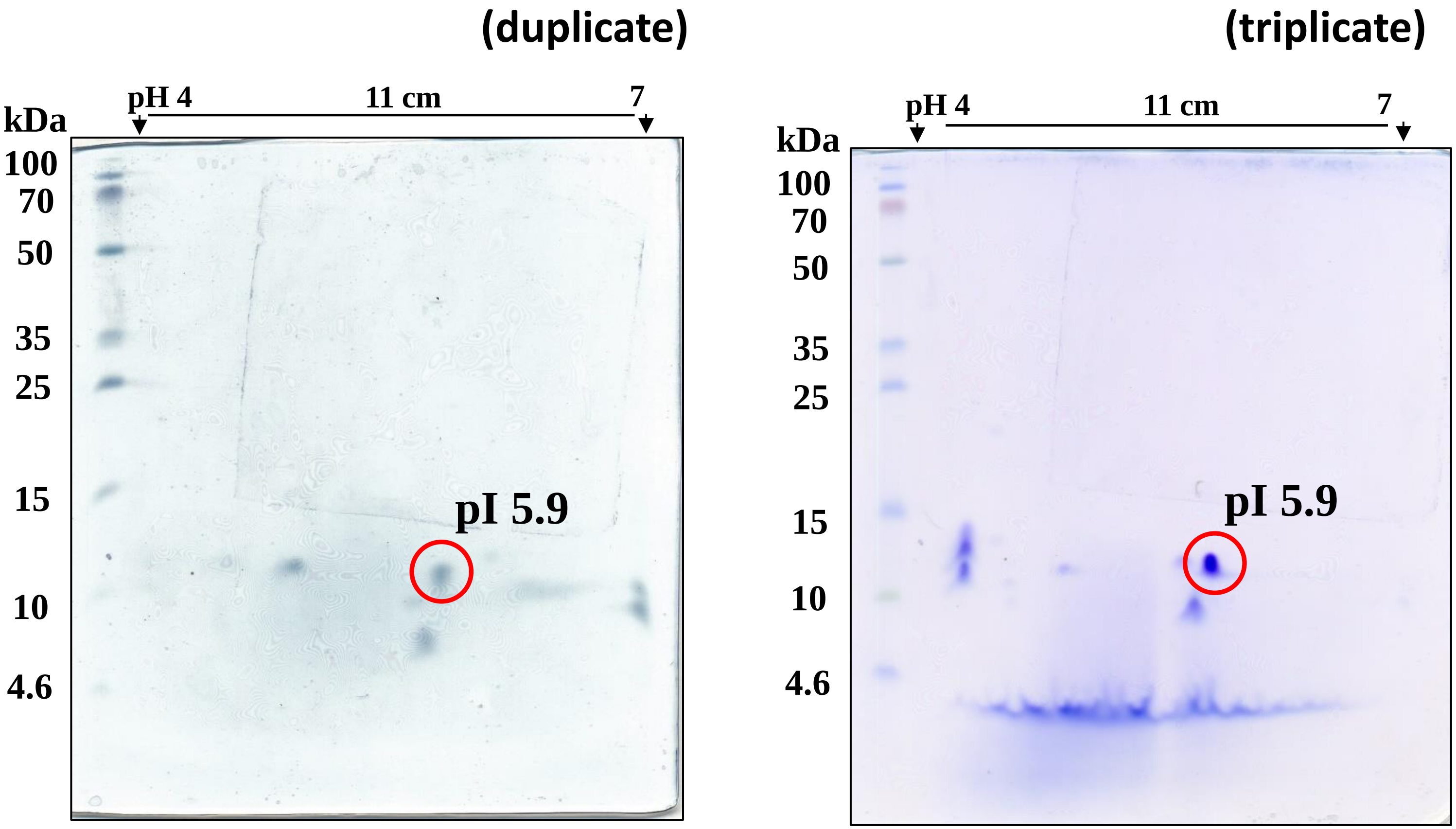

Figure 3A. 2-D gel electrophoresis of PnBBI under reducing conditions

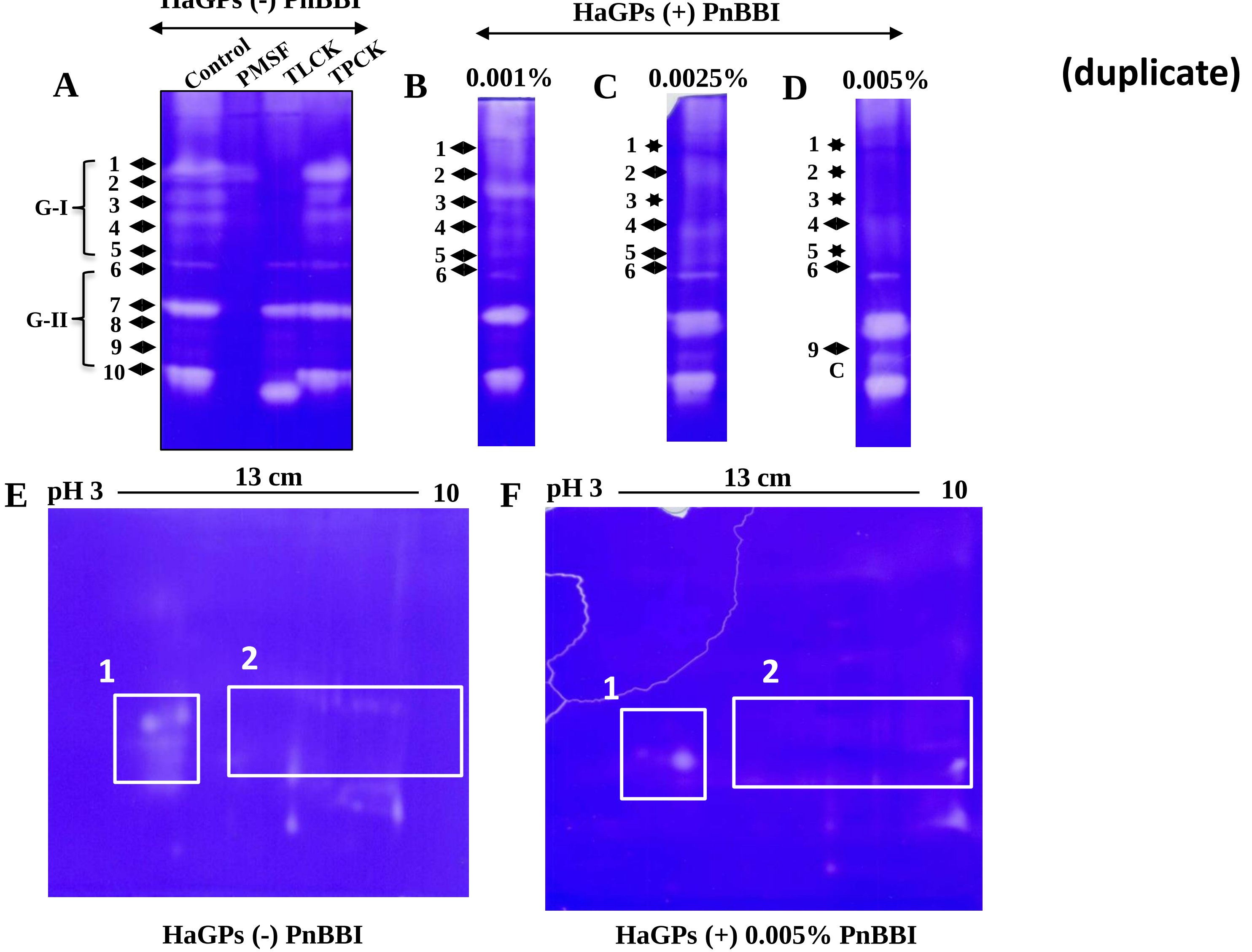

Figure 8. Comparative Zymography of HaGPs from larvae fed on PnBBI. Casein Native-PAGE zymogram of HaGPs extracted from larvae (day9) fed on diet with **(A)** No PnBBI; **(B)** 0.001% PnBBI; **(C)** 0.0025% PnBBI and **(D)** 0.005% PnBBI. Differential 2-DE zymography of HaGPs from larvae fed on **(E)** control diet and **(F)** 0.005% PnBBI supplemented diet.

(duplicate)

Purification step	Total protein (mg)	Total activity (TI units)	Yield recovery (%)	Specific activity (TIU/mg)	Purification fold change
Crude extract	933.8	7666.6	100	8.33	1
$(NH_4)_2SO_4$ fractionation (20-60%)	299	3445	44.93	11.53	1.38
Trypsin-Sepharose 4B	3.6	1260	16.43	350	42.1
Sephadex G-50	0.559	780	10.17	1395	167

(triplicate)

Purification step	Total protein (mg)	Total activity (TI units)	Yield recovery (%)	Specific activity (TIU/mg)	Purification fold change
Crude extract	1344	10500	100	7.81	1
$(NH_4)_2SO_4$ fractionation (20-60%)	880	9163	87.2	10.41	1.33
Trypsin-Sepharose 4B	10	2500	23.8	250	32
Sephadex G-50	1	999	9.5	1000	128

Table 1. Purification of PnBBI from mature seeds of peanut interspecific advanced variety 4368-1.
