# Supplementary material for: Response of Midgut Trypsin- and Chymotrypsin-Like Proteases of Helicoverpa armigera Larvae Upon Feeding With Peanut BBI: Biochemical and Biophysical Characterization of PnBBI
Source: Front Plant Sci. 2020 Mar 24;11:266. doi: 10.3389/fpls.2020.00266 (PMC7105688; doi:10.3389/fpls.2020.00266)
Supplement: Supplementary file 5 [file Table_1.DOCX]

**Supplementary Table 1**

**List of primers used for quantitative real-time PCR analysis.** The primers for all five trypsin-like (*HaTry*1, 4, 6, 7 and 8) and three chymotrypsin-like (*HaChy*1, 2 and 4) protease genes of *H. armigera* including housekeeping gene sequence of ribosomal protein S18 were adopted from Chougule et al. (2005), Chikate et al. (2013) and Mahajan et al. (2013).

| **Name** | **GenBank ID** | **Forward/**  **Reverse** | **Sequence** | **No. of**  **bases** |
| --- | --- | --- | --- | --- |
| *HaTry1* | EU982841 | F | GAGGACACAGATGTGGAGGGG | 21 |
|  |  | R | GAACACACGGAATTCAGCCACG | 22 |
| *HaTry4* | EF600059 | F | GTGCTACCCCTTCTGATTC | 19 |
|  |  | R | AACTTGTCGATGGAGGTGAC | 20 |
| *HaTry6* | Y12276 | F | CCATCGCCGGTGCCAACTA | 19 |
|  |  | R | CTGAACGTGACGCAACTGCTC | 21 |
| *HaTry7* | Y12271 | F | CAGAGGATTGTGGGTGGTTCG | 21 |
|  |  | R | GCGGTGAGGATAGCCCTGTT | 20 |
| *HaTry8* | Y12286 | F | GGGCTACTGGTGCCTTCAACG | 21 |
|  |  | R | CAGAGTCATACACGTCACCGACG | 23 |
| *HaChy1* | Y12287 | F | GCA CCA GAC TGA ACA CCG CTA G | 22 |
|  |  | R | GCG ATG TTG CCA GAA GTA GCA ACG | 24 |
| *HaChy2* | EU325550 | F | GACTTGTCAGGTGGCCAGGCTG | 22 |
|  |  | R | GCGATTCTGGTACCGCCGGAGAAC | 24 |
| *HaChy4* | Y12273 | F | CACCATCTTCATCTTCCAATCCGTGTGC | 28 |
|  |  | R | GTGTTGATACGAGTACCACCGAAGAAC | 27 |
